# Whole-genome resequencing of 495 *Pyrus* accessions provides insights into the genetics of agronomic traits and evolutionary history of pear

**DOI:** 10.1093/hr/uhag042

**Published:** 2026-03-02

**Authors:** Simeng Zhang, Ying Zhang, Jinpeng Bi, Jiayu Xu, Luming Tian, Xingguang Dong, Yang Yu, Wei Heng, Dan Qi, Hongliang Huo, Chao Liu, Ruiqing Pan, Xiang Yang, Chenxi Xu, Yufen Cao

**Affiliations:** Research Institute of Pomology, Chinese Academy of Agricultural Sciences, Xingcheng 125100, China; Research Institute of Pomology, Chinese Academy of Agricultural Sciences, Xingcheng 125100, China; Berry Genomics Corporation, Beijing 100015, China; Research Institute of Pomology, Chinese Academy of Agricultural Sciences, Xingcheng 125100, China; Research Institute of Pomology, Chinese Academy of Agricultural Sciences, Xingcheng 125100, China; Research Institute of Pomology, Chinese Academy of Agricultural Sciences, Xingcheng 125100, China; Beijing Academy of Agriculture and Forestry Sciences, Beijing 100097, China; School of Horticulture, Anhui Agricultural University, Hefei 230036, China; Research Institute of Pomology, Chinese Academy of Agricultural Sciences, Xingcheng 125100, China; Research Institute of Pomology, Chinese Academy of Agricultural Sciences, Xingcheng 125100, China; Research Institute of Pomology, Chinese Academy of Agricultural Sciences, Xingcheng 125100, China; Berry Genomics Corporation, Beijing 100015, China; Research Institute of Pomology, Chinese Academy of Agricultural Sciences, Xingcheng 125100, China; Research Institute of Pomology, Chinese Academy of Agricultural Sciences, Xingcheng 125100, China; Research Institute of Pomology, Chinese Academy of Agricultural Sciences, Xingcheng 125100, China

## Abstract

Pear (*Pyrus* L.) is a fruit tree of global commercial importance. Its genetic relationships, evolutionary history, dissemination routes, and genetic determinants of most agronomic traits remain to be elucidated. We conducted whole-genome resequencing of 495 *Pyrus* accessions. Phylogenetic and demographic analyses resolved geographic groupings of the accessions, identifying the Yunnan–Guizhou Plateau as the putative dissemination center for cultivated *Pyrus pyrifolia* and *P. bretschneideri*. Identification of two evolutionary bottlenecks provides insights into the population dynamics of pear species. Admixture and introgression analyses revealed both intraspecific and interspecific genetic exchanges, substantiating the complex emergence of cultivated populations. Genome-wide association study (GWAS) identified loci associated with nine crucial agronomic traits, together with eight candidate genes. The GWAS, molecular, and biochemical analyses suggested that *PbeMADS25*, *PbeSPP*, *PbeDHQ-SDH*, *PbeARF2*, *PbePPO*, *PbePIN3*, *PbeCXE*, and *PbeMYB38* participate in the regulation of number of stigmas and number of locules, number of stamens, young leaf color, sepal persistence, astringency, acidity, aroma, and fruit skin color, respectively. Overexpression and metabonomic analysis of *PbeCXE* indicated that it affects the fruit aroma by affecting the balance between ester biosynthesis and substrate consumption. These findings expand our understanding of *Pyrus* evolution and provide a genomic foundation for genetic improvement of agronomic traits.

## Background

Pear (*Pyrus* L.) is a globally important temperate fruit tree that is believed to have originated in western China during the Tertiary period [1]. Following its domestication in Eurasia, two major geographically distinct groups emerged, the Eastern pears and Western pears, comprising more than 20 and 12 to 15 species, respectively [1, 2] *Pyrus communis* (common pear; CP) is primarily grown in Europe, North and South America, North Africa, and Australia, whereas *P. bretschneideri* (white pear [WP]), *P. pyrifolia* (sand pear [SP]), *P. ussuriensis* (Ussurian pear [UP]), and *P. sinkiangensis* (Xinjiang pear [XP]), which are distinguished by morphological characteristics and ecological preferences, are cultivated predominantly in East Asia [3–5]. Previous studies have provided evidence for the independent domestication of Asian and European pears, and that the two recognized subgenera of *Pyrus* have followed independent evolutionary paths, influenced by geographical barriers formed through the uplift of the Tibetan Plateau and increased aridity in Central Asia [6–8]. The domestication process of wild pears in Europe has been reviewed and, in three Mediterranean *P. pyraster* populations, evidence for significant cultivated-to-wild gene flow has been reported [9, 10]. Sand pear is considered to have spread eastward along the Pearl River and Yangtze River valleys. Pear migration occurred from southwest to southeast and from south to north, and *P. ussuriensis* encompasses higher genetic diversity [11, 12]. Chinese sand pear, Chinese white pear, and Japanese pear are derived from the same progenitor of *P. pyrifolia* in China [13]. Given extensive intra- and interspecific hybridization, cultivated pears exhibit complex phylogenetic relationships, particularly among Asian pear populations, and their origin and taxonomy remain controversial [13–16]. Recent studies have suggested more nuanced divergence patterns than previously envisaged [6, 17]. Although earlier studies have proposed models of pear divergence and dissemination [6, 13], the dispersal routes and demographic history of *Pyrus* remain poorly resolved.

To understand how morphology reflects these complex evolutionary histories, phenotypic descriptors remain essential for classification [18]. The number of locules per fruit is of particular importance for *Pyrus* domestication because it has increased from wild species to cultivated forms. This increase led to the historical classification of *Pyrus* into three sections: *Micropyrus*, *Intermedia*, and *Eupyrus* [15]. Sepal persistence has historically been considered a critical taxonomic character, with persistent sepals more frequently observed in *P. communis* and *P. ussuriensis* [4, 14]. The color of the young leaf exhibits considerable variation and is a useful trait for distinguishing pear germplasm resources during blooming [19]. Some traits significantly affect fruit quality, with consumers typically preferring different colors, non-astringent, aromatic, and low-acidity fruit. However, a limited number of genes associated with these traits have been identified to date and such research is ongoing [6, 17, 20–23]. Thus, application of high-resolution methods, such as genome-wide association study (GWAS), is needed to bridge this knowledge gap.

To elucidate the genetic complexity underlying agronomically important traits, such as fruit quality and domestication signatures—previously constrained by the limitations of phenotypic analysis—genomic tools emerged to provide transformative solutions. In 2013, a detailed genetic map for *P. bretschneideri* ‘DangshanSuli’ was assembled and sequenced [24]. In 2019, our research team completed the assembly of the first genome for a wild pear, *P. betulifolia* ‘Shanxi Duli’ and 95.9× coverage of SMRT sequences was achieved [23]. These resources enable genome-wide resequencing, now a standard tool in genetic research. By comparing whole-genome resequencing results with a reference genome sequence, the genetic variation across the entire genome can be detected. Whole-genome resequencing has been widely applied in genetic research on fruit trees. For example, genome resequencing of 26 cultivated and 11 wild loquat accessions showed that wild loquats harbor higher genetic diversity than cultivated loquats. Genes associated with phenotypic traits for fruit quality, fruit size, and leaf color were selected during evolution [25]. Based on a selective-sweep analysis of geographical groups within a core collection of 74 accessions of Chinese plum, 35 loci were selected, and genes involved in regulating pathways, such as flowering, resistance, and flavonoid metabolism, were significantly enriched, revealing the molecular basis of important domestication traits of Chinese plum [26]. A genome-wide analysis of genetic variation in a natural population of 312 sand pear varieties and GWAS analysis of eight fruit-quality traits of pear, including single fruit weight, fruit color, and stone cells, enabled identification of genetic loci associated with stone cells in pear fruit [17] To date, whole-genome resequencing has enabled progress in the gene mining of important traits in pear. However, because the genetic variation of the materials analyzed is limited, the genes underlying numerous traits have not been localized or are at an early stage of localization, and the regulatory mechanisms of many traits remain unclear. Furthermore, the candidate functional genes have not been verified.

In this study, we conducted a genomic variation and demographic analysis on a global collection of 495 *Pyrus* accessions, encompassing wild relatives, landraces, and cultivars. Utilizing phenotypic data recorded over 3 years, we performed GWAS to identify loci associated with key agronomic traits. We identified candidate genes that were potentially targets of selection during domestication. Genomic signatures of selective sweeps were examined to uncover domestication-related genetic variation. The findings provide insights into the divergence, evolutionary history, dissemination, and genetic determinants of critical agronomic traits in *Pyrus*.

## Results

**Figure 1:**
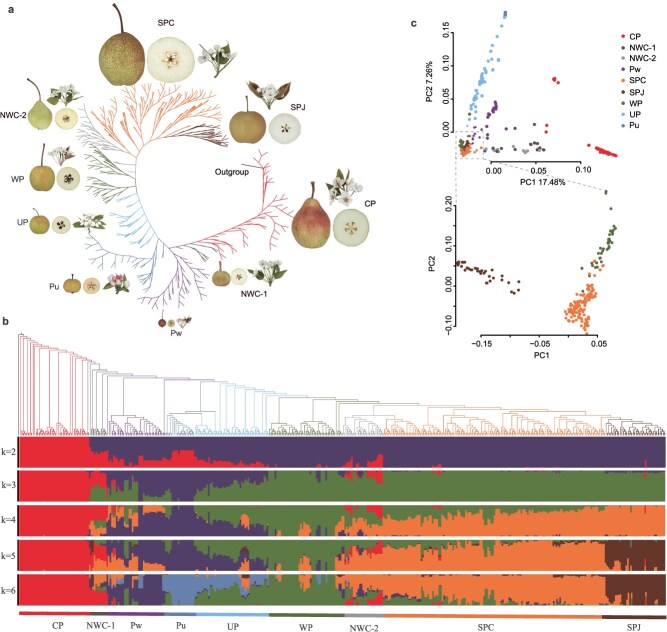
Phylogenetic relationships and population structure of *Pyrus* accessions. **a**, Maximum-likelihood phylogenetic tree for 407 *Pyrus* accessions constructed using whole-genome SNPs with 100 nonparametric bootstrap replicates. **b**, Upper panel: PCA scatterplot of 407 *Pyrus* accessions; Lower panel: PCA scatterplot of the WP, SPC, and SPJ accessions (boxed region in the upper panel). **c**, Population structure of 407 *Pyrus* accessions based on a model-based clustering analysis with ancestry kinship (*k*) of 2 to 6.

**Genomic variation and population structure**. Genomic resequencing of 495 diverse *Pyrus* accessions (Supplementary Table S1) generated 4.13 Tb of high-quality sequencing data, with an average mapping depth of 10.72× against the pear reference genome^[23].^ Using this dataset, we identified 11 031 864 high-quality single-nucleotide polymorphisms (SNPs; Supplementary Tables S2 and S3). Eighty-eight accessions of unknown origin or those classified as improved varieties in China were excluded from the analysis, retaining 407 accessions for maximum-likelihood phylogenetic analysis, principal component analysis (PCA), and population structure analysis (Fig. 1a, Supplementary Fig. S1, Supplementary Table S4). The geographic descriptors assigned to groups of accessions reflect the predominant origin within each genetic group and are intended solely for descriptive purposes, as instances of introgression and germplasm exchange have resulted in occasional mixed geographic provenances.

The wild *Pyrus* species were classified into two main genetic groups: Pu (wild *P. ussuriensis*) and Pw (comprising *P. betulifolia* [Pb], *P. calleryana* [Pc], *P. xerophila* [Px], *P. phaeocarpa* [Pp], *P. hopeiensis* [Ph], and *P. serrulata* [Ps]). Pu and Pw represented distinct wild genetic lineages likely shaped by long-term natural selection and were genomically differentiated from cultivated accessions. The pronounced genetic isolation and cold-adaptive genome of Pu support its status as a relatively independent wild progenitor lineage. Cultivated pear accessions were grouped into seven geographically distinct groups, comprising CP (*P. communis*) from Europe, NWC-1 (*P. sinkiangensis*) and NWC-2 (*P. bretschneideri* and *P. sinkiangensis*) from Northwest China, UP (cultivated *P. ussuriensis*) from Northeast China, WP (*P. bretschneideri*) from Yan Mountain, SPC (Chinese *P. pyrifolia*) from Yan Mountain in the north, the Yunnan–Guizhou Plateau, and the West Sichuan Plateau in the west, and SPJ from Japan. NWC-1 and NWC-2 were genetically admixed populations derived both from European and Asian pear species, and were further distinguished by their distribution in different regions of Northwest China. The SPC group was subdivided into four geographical subgroups: SPC-YG (Yunnan–Guizhou Plateau), SPC-SW (Sichuan Basin and West Sichuan Plateau), SPC-SE (Southeastern Hills), and SPC-NC (North China) (Supplementary Fig. S3d).

When *k* = 6, the clusters maximized the marginal likelihood (Fig. 1b, Supplementary Fig. S2). CP formed a genetically homogeneous monophyletic clade and as the k value increased from 2 to 6, the genetic distinctions between the groups became more evident. At *k* = 4, WP was distinguished from SP with clear genetic introgression, particularly between WP and SPC-NC. SPJ accessions clustered independently, showing a unique genetic background at *k* = 5 which was closely related to SPC-SE. In contrast to SPC, genetic introgression between WP and SPJ was rare. At *k* = 6, the genetic distinction between Pw and Pu became clear, and obvious introgression between Pu and WP. Both NWC-1 and NWC-2 were genetically admixed populations of European and Asian pears. Most accessions in NWC-1, primarily from Xinjiang and classified as XP, were more introgressed from CP, while those in NWC-2, distributed across Gansu, Qinhai, and Xinjiang, and morphologically classified as SPC, WP, or XP, were genetically more closely related to SPC-YG.

The ADMIXTURE analysis revealed progressive genetic stratification across *K*-values (Fig. 1b). At *K* = 3, three genetically distinct populations corresponding to wild pears, cultivated European pears, and cultivated Asian pears were resolved, supporting their classification as independent evolutionary units. With increase in *K* value from 2 to 6, the genetic distinctions between the groups became increasingly evident. At *K* = 4, WP was distinguished from SP, with pronounced genetic introgression, particularly between WP and SPC-NC. At *K* = 5, SPJ accessions were grouped separately, revealing a unique genetic background that was closely related to SPC-SE. In contrast to SPC, genetic introgression between WP and SPJ was rare. At *K* = 6, Pw and Pu were clearly distinguished, and introgression between Pu and WP was indicated. Both NWC-1 and NWC-2 were genetically admixed populations of European and Asian pears. Most accessions in NWC-1, primarily those from Xinjiang and classified as XP, were more extensively introgressed with CP. The NWC-2 accessions, which are distributed in the Gansu, Qinhai, and Xinjiang provinces, and were morphologically classified as SPC, WP, or XP, were genetically more closely related to SPC-YG.

The genetic relationships inferred from the maximum-likelihood phylogenetic tree were supported by the PCA results (Fig. 1c). CP and Pu formed distinct groups, while three groups of Asian crisp-fleshed accessions (SPJ, WP, and SPC) were grouped together. In the subsequent PCA of these three groups, WP partially overlapped with SPC, supporting their close genetic relationship. SPJ was separated from SPC and WP on the first principal component, indicating that SPJ is a genetically distinct population. Both NWC-1 and NWC-2 exhibited broad genetic diversity, and were genetically connected with CP and Asian SPC and WP, consistent with their origin by interspecific hybridization. Notably, the elevated nucleotide diversity in NWC-1 and NWC-2 is consistent with the known breeding history of modern cultivated pears, in which germplasm exchange between Eastern and Western lineages has been common. Although genetically distinct, NWC-1 and NWC-2 each represent outcomes of parallel domestication and genetic improvement in different regions, resulting in comparable levels of admixture-derived heterogeneity.

UP showed genetic similarity with the crisp-fleshed WP and the soft-fleshed Pu, supporting hybridization between *P. ussuriensis* and *P. bretschneideri* [13]. High levels of admixture were detected in some classification-ambiguous cultivars. For example, ‘Pinguoli’ from Yanbian, which was resolved in the UP group in the phylogenetic analysis, had a complex genetic background involving WP, SPC, SPJ, and Pu. Similarly, ‘Korla Pear’, a commercial cultivar from Xingjiang, was placed in the NWC-2 group and showed genetic admixture from CP, WP, and SPC, and also a close genetic relationship with ‘Red Huoba’ from Yunnan and ‘Donghuang’ from Xinjiang (Supplementary Table S1).

**Figure 2:**
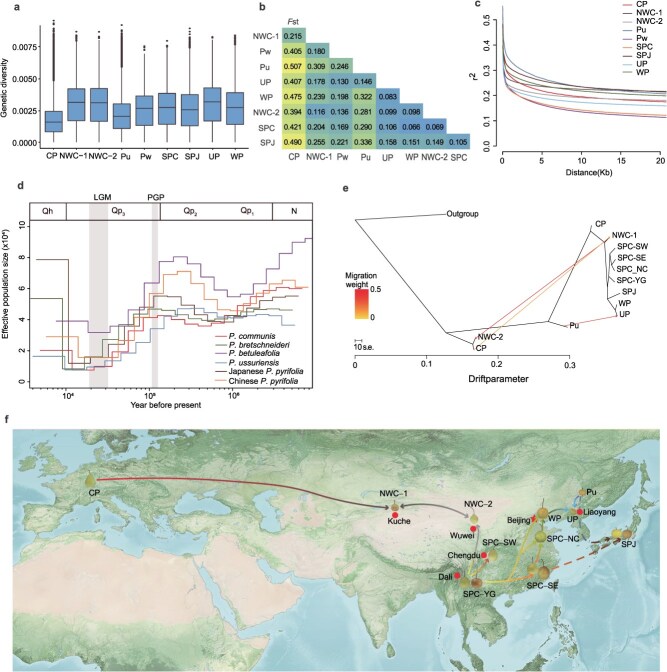
Genetic diversity, linkage disequilibrium, demographic history, and putative dissemination routes for *Pyrus*. **a**, Boxplot of the nucleotide diversity (*π*) of *Pyrus* groups. **b**, Divergence of the *Pyrus* groups based on *F*_st_ values. The summary statistics were calculated using a 1 Mb window size and a 1 Mb step size. **c**, Linkage disequilibrium among the *Pyrus* groups. **d**, Demographic history of *Pyrus* illustrating the effective population size (*N*_e_; inferred by multiple sequentially Markovian coalescent analysis) of *Pyrus* species. Coordinates are logarithmically scaled. **e**, Detection of gene flow among the *Pyrus* groups and subgroups of SPC based on a TreeMix analysis. Arrows represent the direction of dispersal. The horizontal branch length is proportional to the amount of genetic drift that occurred on the branch. The scale bar represents 10 times the average standard error of the entries in the sample covariance matrix. **f**, Putative dissemination routes of *Pyrus*. (Fig. 1a), population genetic analyses (Fig. 1b and c), genetic differentiation (Fig. 2a), demographic history (Fig. 2d), and gene flow (Fig. 2e). The multicolored dashed arrows indicate probable dispersal events. The map was generated using GIS 10.2. Qh, Quaternary Holocene; Qp_1_, early Quaternary Pleistocene; Qp_2_, mid-Quaternary Pleistocene; Qp_3_, late Quaternary Pleistocene; N, Neogene; LGM, Last glacial maximum; PGP, penultimate glacial period.

**Divergence, demographic history, and dissemination routes**. The fixation index (*F*_st_), nucleotide diversity (*π*), and linkage disequilibrium (LD) decay were estimated for the *Pyrus* population (Fig. 2a–c, Supplementary Tables S5 and S6). The nucleotide diversity across all accessions was 6.22 × 10^−3^, with Asian pear groups exhibiting higher nucleotide diversity (π = 1.96 × 10^−3^ to 2.74 × 10^−3^) compared with the CP group (π = 1.61 × 10^−3^). The CP group showed the most rapid LD decay (1.5 kb). The present results support previous findings [6] that European and Asian pears represent two independent domestication groups (*F*_st_ ≥ 0.215). SPC and SPJ showed similar nucleotide diversity (*π* = 2.43 × 10^−3^ and 2.37 × 10^−3^, respectively), with SPC exhibiting more rapid LD decay (2.5 kb) compared with that for SPJ (8.1 kb) (Supplementary Tables S5 and S6). This pattern is consistent with the broad distribution of Chinese *P*. *pyrifolia* and the effects of inbreeding in geographically isolated Japanese *P*. *pyrifolia*. For the two genetically admixed groups from Northwest China, NWC-1 and NWC-2, nucleotide diversity was higher (*π* = 2.70 × 10^−3^ and 2.73 × 10^−3^, respectively) and LD decay was more gradual (45.2 and 8.4 kb, respectively), indicating that interspecific hybridization contributed to the higher genetic diversity of these populations. In contrast, the nucleotide diversity of wild Pu was lower (*π* = 1.96 × 10^−3^), which suggests a strong bottleneck occurred during the dispersal of *Pyrus* to Northeast China. Genetic divergence among the four SPC subgroups (*F*_st_ = 0.048–0.089) was low and nucleotide diversity was limited, supporting the close genetic relationships among these subgroups (Supplementary Fig. S3a–c, Supplementary Tables S7 and S8).

To investigate the evolutionary history of pear, we performed a multiple sequentially Markovian coalescent (MSMC) analysis to examine the effective population size (*N*_e_) dynamics for several *Pyrus* species, comprising *P. communis*, *P. bretschneideri*, *P. betulifolia*, *P. ussuriensis*, and *P. pyrifolia* from both China and Japan (Fig. 2d). Significant population bottlenecks were indicated during the Penultimate Glacial Period (130–113 ka) and the Last Glacial Maximum (LGM; 33–19 ka), reflecting the severe climatic challenges that these species faced. Post-LGM, with the advent of the Holocene, population recovery and gradual expansion were indicated, likely driven by less extreme climatic conditions. Of particular interest, *P. betulifolia* exhibited the least pronounced population declines during the LGM, suggesting that it showed greater resilience to extreme cold. In contrast, the major cultivated species, *P. communis*, *P. bretschneideri*, and *P. pyrifolia*, experienced more severe population contractions during the LGM, but subsequently showed marked recovery and expansion during the Holocene. This recovery is likely correlated with the rise of human agricultural practices and selective breeding, which may have played a pivotal role in enhancing the population growth and dispersal of cultivated pear.

We performed an ABBA/BABA analysis to detect genetic introgression among Asian pear genetic groups. Relatively strong introgression was indicated between SPC and SPJ, UP and WP, and WP and SPC, respectively (Supplementary Table S9). TreeMix analyses detected three gene-flow events among the *Pyrus* groups (Fig. 2e, Supplementary Fig. S4). The migration edge inferred from NWC-2 to NWC-1 is consistent with an NWC-2-like ancestry contributing to NWC-1. Similarly, the edge from CP to NWC-2 supports the contribution of a CP-like ancestry to NWC-2. In addition, gene flow inferred from Pu to UP is consistent with the domestication and cultivation of *P. ussuriensis*. These patterns agree with the model-based clustering results (Fig. 1c).

The Yunnan–Guizhou Plateau was the putative dissemination center for cultivated *P. pyrifolia* and *P. bretschneideri*, from where *P. pyrifolia* spread northward to form SPC-SW in the Sichuan Basin and West Sichuan Plateau, and spread eastward to form SPC-SE in the Southeastern Hills [6]. After spreading eastward across the Yunnan–Guizhou Plateau, dissemination northeastward formed *P. bretschneideri* on Yan Mountain coinciding with eastward dispersal, together with the northward dispersal of SPC-SE, to form SPC-NC in North China [13]. Hybridization between *P. bretschneideri* and *P. ussuriensis* gave rise to the cultivated forms of *P. ussuriensis* in Northeast China; subsequent spread occurred northward to Gansu and then westward to the Hexi Corridor and further westward to Xingjiang where hybridization with *P. communis* cultivars from Europe resulted in the formation of NWC-2 and NWC-1, which included *P. sinkiangensi*s [12]^.^ Finally, SPC-SE probably spread eastward to Japan, together with the ancestral component of Pu from Northeast China, to form SPJ [14](Fig. 2f).

**Genome-wide selection of domestication traits**. We searched for signatures of selection in the pear genome by comparing the Pb and Pc accessions in the Pw group (predominantly 2–3 locules) with six groups that evolved 4 to 7 locules (CP, SPC, SPJ, WP, UP, and Pu). The overlap between the *F*_st_ values (top 5% for the entire genome) and the cross-population composite likelihood ratio (XP-CLR) values (top 10%) was used to identify candidate selective sweeps. An average of 1512 regions with an average of 4170 genes were selected following the six comparisons of the *F*_st_ value. An average of 4212 regions with an average of 10 058 genes were selected after the six comparisons of the XP-CLR value. Based on the overlap between *F*_st_ and XP-CLR values, we identified 2900 genes in at least four of the six comparisons (Supplementary Fig. S6b). The KEGG pathways enriched among the selected genes included ‘alpha-Linolenic acid metabolism’ (mdm00592), ‘Tyrosine metabolism’ (mdm00350), and ‘Phenylalanine metabolism’ (mdm00360). These pathways are associated with fruit quality [27–29] and might suggest that fruit quality traits were selected during domestication (Supplementary Fig. S5, Supplementary Table S10).

**Figure 3:**
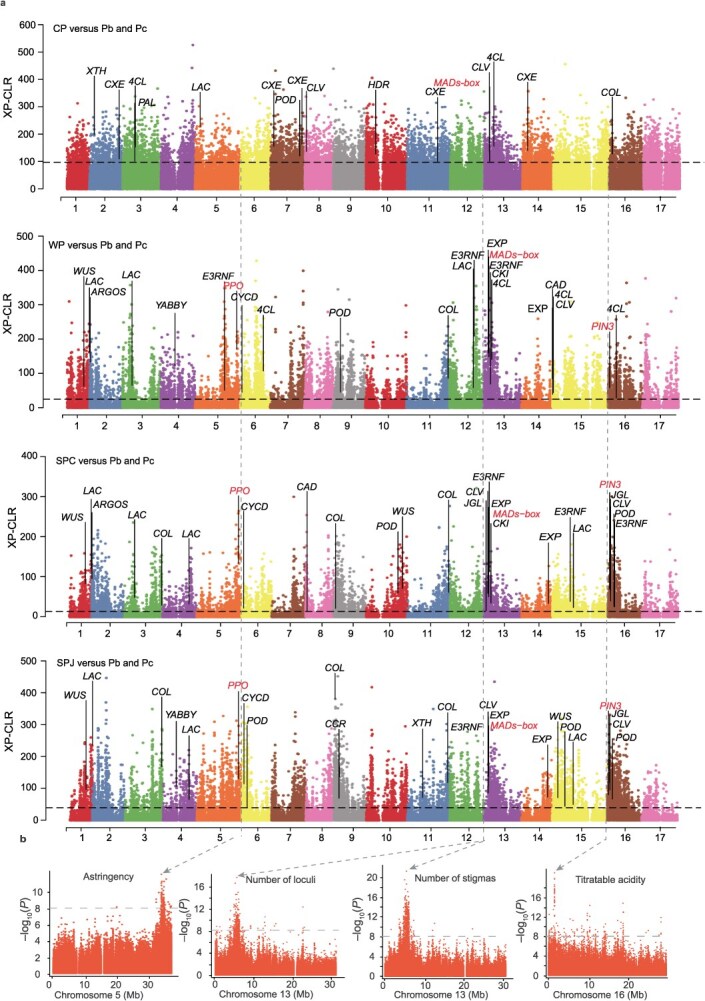
Identification of selective sweeps associated with domestication traits in crisp-fleshed Asian pear accessions. **a**, Genome-wide selective-sweep signals detected by comparing WP, SPC, and SPJ with Pw (only Pb and Pc) based on XP-CLR values. The horizontal dashed line indicates the cutoff, with the highest being 10%. **b**, Manhattan plots of the GWAS signals overlapping with the identified selective sweeps for different traits. The horizontal dashed line indicates the significance threshold (*P* < 1 × 10^−8^, Bonferroni correction).

MADS-box genes associated with the number of stigmas and the number of locules were selected in the comparisons with four highly evolved groups (SPC, SPJ, WP, and CP), which was consistent with the GWAS results. *PIN3* and *PPO*, which are associated with fruit acidity and fruit astringency, respectively, were selected in the comparisons with three Asian crisp-fleshed groups (SPC, SPJ, and WP), which was also consistent with the GWAS results (Fig. 3a and b, Supplementary Figs S6a, S7, and  S8, Supplementary Table S11). *EXP* and *XTH*, which are associated with flower size in carnation [30], were selected, but differences among the six comparisons were evident, with *EXP* detected in SPC and WP, *XTH* in CP and Pu, and *EXP* and *XTH* in SPJ and Pu, which might be consistent with the increase in flower size during evolution. The *COL* gene, which is associated with the photoperiodic control of flowering [31], was selected in all comparisons, with the exception of the comparison with Pu (the earliest flowering group). *ARGOS* and *JGL*, which are associated with leaf size in soybean [32], were selected in all comparisons, except for the comparison with CP (which has relatively small leaves). *WUS* and *CLV*, which are associated with fruit size in tomato [33], were selected in all comparisons. *CKI*, *CYCD*, *E3RNF*, and *YABBY*, which influence fruit size in pear [6, 17], were selected only in the comparisons with the Asian pear groups. Some of the stone cell-related genes [34] that were selected in all comparisons might help to explain the textural differences among the cultivated populations. The *CXE* gene, which enhances the aroma of strawberry and white-fleshed pitaya fruit [35, 36], as well as the *HDR* gene, which is associated with fruit aroma in grape [37], were selected only in the comparisons with the intensely flavored and aromatic CP group (Fig. 3a, Supplementary Figs S6a, S7, and  S8).

**Figure 4:**
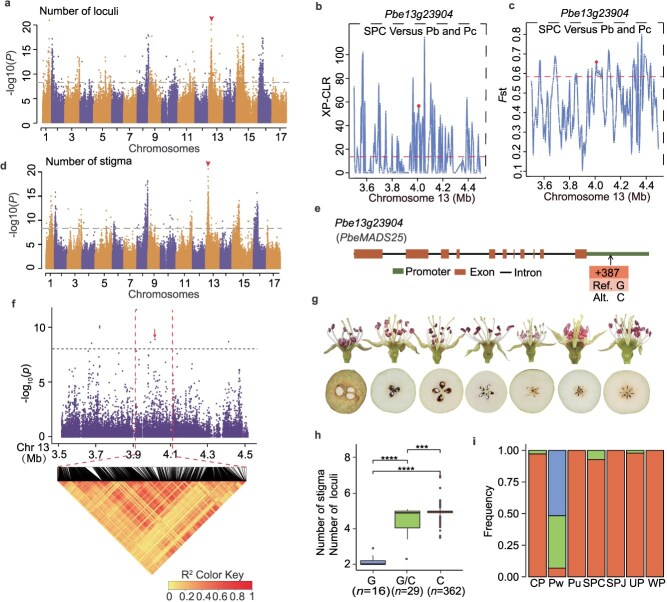
GWAS of the number of stigmas and number of locules per flower and identification of *PbeMADS25* as the candidate gene. **a**, Manhattan plot of GWAS −log_10_ (*P* values) indicating the strength of SNP association with the number of locules. **b**, *Pbe13g23904* (*PbeMADS25*) based on cross-population composite likelihood ratio (XP-CLR) analysis. The candidate gene is indicated by a red dot. **c**, *Pbe13g23904* (*PbeMADS25*) based on *F*_st_ analysis. **d**, Manhattan plot of GWAS −log_10_(*P*-values) indicating the strength of SNP association with the number of stigmas. **e**, Gene structure of *Pbe13g23904* (*PbeMADS25*)*.* Ref., reference; Alt., alternate. **f**, Local Manhattan plot (top) and linkage disequilibrium heatmap (bottom) surrounding the peak on chromosome 13. In **a, d**, and **f**, the horizontal dashed line indicates the significance threshold [−log_10_(*P*) = 8.34]. **g**, Number of stigmas and number of locules in pear. **h**, Box plot of the number of stigmas and number of locules according to the genotype of the SNP (Chr13_4,015650). In the box plots, the center line denotes the median, the box limits are the first and third quartiles, and the whiskers indicate the data range. The significance of differences was analyzed using a two-tailed Student’s *t*-test. **i**, Variation in *Pbe13g23904* (*PbeMADS25*) haplotype frequency among groups of pear accessions.

**GWAS analysis for clarity of plant architecture traits**. Two characters, namely, number of stigmas and number of locules, were designated domestication traits associated with fruit size (Fig. 4g) [15, 38]. The mapping results were highly similar for these two traits (Fig. 4a and d). The GWAS analysis identified one SNP (Chr13:4015650) that was significantly associated with both traits (Fig. 4f). The accessions with the reference GG sequence had fewer stigmas and locules than the accessions with the alternate CC sequence (Fig. 4h). The associated signal was located in the *Pbe13g23904* (*PbeMADS25*) promoter region (Fig. 4e, Supplementary Table S12). The *PbeMADS25* gene encodes a MADS-box transcription factor that reportedly influences floral development and flowering [39]. *PbeMADS25* was a commonly selected gene in SPC (Fig. 4b and c). Most of the *Pyrus* sect. *Micropyrus* accessions (2–3 locules; Pb and Pc) carried the G/G allele, the *Pyrus* sect. *Intermedia* accessions (3–4 locules; *P. xerophila*, *P. phaeocarpa*, *P. hopeiensis*, and *P. serrulata*) mainly carried the G/C allele, and the *Pyrus* sect. *Eupyrus* accessions (4–7 locules: *P. bretschneideri*, *P. pyrifolia*, *P. ussuriensis*, and *P. communis*) primarily carried the C/C allele (Fig. 4i).

The number of stamens per flower is correlated with pollination and affects fruit set in pear. The GWAS analysis detected one candidate region for this trait on chromosome 15 (Supplementary Fig. S9a). The candidate region included one candidate gene, *Pbe15g04055* (*PbeSPP*), which encodes a signal peptide peptidase. In Arabidopsis, *AtSPP* is required for male gametophyte development and pollen maturation [40]. A significantly associated signal was localized to a nonsynonymous SNP (Chr15_5,877 006; C to A) within this gene (Supplementary Fig. S9b and c). The accessions carrying the A/A (alternate) allele had significantly more stamens per flower than the accessions carrying the C/A or C/C alleles (Supplementary Fig. S9d). The A/A genotype was exclusive to SPJ and SPC, which had a greater number of stamens per flower than the other cultivated accessions and wild species (Supplementary Fig. S9e) [41].

**Figure 5:**
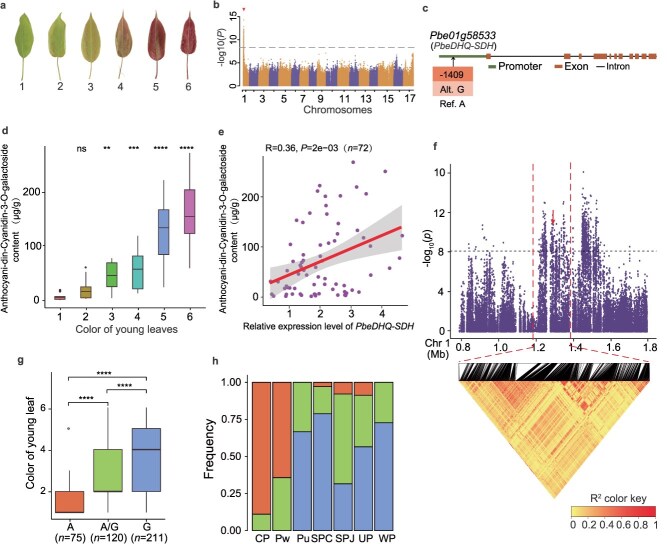
GWAS of the young leaf color and identification of *PbeDHQ-SDH* as the candidate gene. **a**, Phenotypic states of the young leaf color. 1, Green; 2, green slightly tinged with red; 3, green tinged with red; 4, red tinged with green; 5, red slightly tinged with green; 6, red. **b**, Manhattan plot of GWAS −log_10_(*P*-values) indicating the strength of SNP association with young leaf color. **c,** Gene structure of *Pbe01g58533* (*PbeDHQ-SDH*). **d**, Multiple comparisons of anthocyanidin-cyanidin-3-*O*-galactoside content. **e**, Correlation analysis of anthocyanidin-3-*O*-galactoside content and expression level of the candidat*e* gene *PbeDHQ-SDH* in young leaves differing in color. **f**, Local Manhattan plot (top) and linkage disequilibrium heatmap (bottom) surrounding the peak on chromosome 1. **g,** Box plot of young leaf color in relation to the genotype of the SNP (Chr1_1,290 951). **h**, Variation in *Pbe01g58533* (*PbeDHQ-SDH*) haplotype frequency among groups of pear accessions.

The color of the young leaf is a highly variable ornamental trait (Fig. 5a) [19]. The GWAS of this trait revealed one significantly continuous peak corresponding to a region on chromosome 1 (Fig. 5b and f). One gene, *Pbe01g58533* (*PbeDHQ-SDH*), which encodes a bifunctional 3-dehydroquinate dehydratase (DHQ-SDH) and was mapped to the 1.23–1.31 Mb interval, was identified in this region. A previous study showed that DHQ-SDH supplies the substrates for the anthocyanin biosynthetic pathway, thereby regulating the color of young ‘Zijuan’ tea leaves [42]. We measured the anthocyanin content in young leaves of pear accessions that varied in color and determined that the anthocyanidin-cyanidin-3-*O*-galactoside content significantly increased with increase in the intensity of red coloration (Fig. 5d). This result was similar to findings reported for apple, where cyanidin 3-*O*-galactoside is the primary component responsible for the red pigmentation in the skin of apple fruit [43]. Quantitative real-time PCR (qRT-PCR) analysis revealed that *PbeDHQ-SDH* expression was higher in accessions with red young leaves (Supplementary Fig. S10). Moreover, the expression level of *PbeDHQ-SDH* was significantly positively correlated with the cyanidin 3-*O*-galactoside content (*r* = −0.36, *P* = 2e-03, two-tailed Student's *t*-test) in a panel of 72 pear accessions (Fig. 5e). These results suggested that SDH may play a role in promoting the accumulation of cyanidin-3-*O*-glucoside, contributing to the red coloration in the young leaves of pear. The variability in young leaf color was strongly associated with an SNP (G/A) in the *PbeDHQ-SDH* promoter region (Fig. 5c)*.* Most accessions with green young leaves carried the A/A allele, whereas the accessions with red young leaves carried the G/G allele (Fig. 5g). The frequency of the A/A genotype was significantly higher in the CP group than in the other groups of pear accessions (Fig. 5h).

The sepal persistence affects the internal and external quality of pear fruit. Pear fruit with deciduous sepals (usually termed ‘female pears’) have a more desirable flavor compared with that of fruit with persistent sepals (‘male pears’). The SNP most significantly associated with this trait was previously detected on chromosome 6 [20] (Supplementary Fig. S11a and c). More precisely, the SNP was detected in the exon of *Pbe06g51770* (*PbeARF2*), which encodes an auxin response factor 2 (ARF2) (Supplementary Fig. S11b). In Arabidopsis, *AtARF2* regulates floral organ abscission [44]. Most pear accessions with persistent sepals carried the C/T allele, whereas the accessions with deciduous sepals carried the T/T allele (Supplementary Fig. S11d). Almost all of the accessions in the CP, Pu, and UP groups with persistent sepals carried the C/T allele (Supplementary Fig. S11e).

**GWAS of fruit characteristics**. The considerable interest in astringency is due to its important effects on fruit quality and human health. Fruit astringency is primarily associated with tannins and other polyphenolic compounds [45]. Previous research has indicated that polyphenol oxidase (PPO) from sand pear can decrease the astringency of black tea leaves [46]. Among the 89 astringency-associated SNPs identified in the present study, 53 (60%) were located on chromosome 5 with strong signal (Supplementary Fig. S12a). We focused on the 34.67- to 34.75-Mb interval containing six candidate genes (*Pbe05g05999*–*Pbe05g06004*).

A qRT-PCR analysis showed that the expression level of *Pbe05g06002* (*PbePPO*) was higher in the mature fruit of astringent pear accessions than in the mature fruit of non-astringent pear accessions (Supplementary Fig. S13). Furthermore, *PbePPO* was a commonly selected gene in SPC (Supplementary Fig. S12b and c). A nonsynonymous SNP (Chr5_34,675 707; G/C) was detected in the conserved domain (Supplementary Fig. S12d, e). The majority of non-astringent accessions in the SPC, SPJ, and WP groups carried the C/C allele, whereas the G/G allele was carried in the astringent accessions in the CP and PU groups (Supplementary Fig. S12f and g).

Acidity strongly influences fruit flavor. The present GWAS results suggested that fruit acidity is a polygenic trait, with many marker–trait associations distributed throughout the genome. One significant peak corresponded to a region on chromosome 16 (Supplementary Fig. S14a). One gene (*Pbe16g31218*; *PbePIN3*) encoding a membrane protein (auxin efflux carrier 3) was detected in this region. The corresponding gene in peach controls fruit acidity [47]. Notably, *PbePIN3* was more highly expressed in mature acidic fruit than in mature nonacidic fruit (Supplementary Fig. S15). In addition, *PbePIN3* was a commonly selected gene in SPC (Supplementary Fig. S14b, c). The variability in fruit acidity was strongly associated with an SNP (G/A) in *PbePIN3* (base pair position 1307) (Supplementary Fig. S14d and e)*.* The accessions carrying the A/A allele had a higher titratable acid content than the accessions carrying the G/G or G/A alleles (Supplementary Fig. S14f). The G/G allele was not detected in the Pu group (extremely acidic fruit) (Supplementary Fig. S14g). The transient overexpression of *PbePIN3* in ‘Whangkeumbae’ pear fruit resulted in a significant increase in malic acid accumulation, reaching 2103.96 μg/g compared with 1966.57 μg/g in the control (Supplementary Fig. S14h and i). This finding indicates that *PbePIN3* may play a critical role in regulating organic acid metabolism in pear fruit.

**Figure 6:**
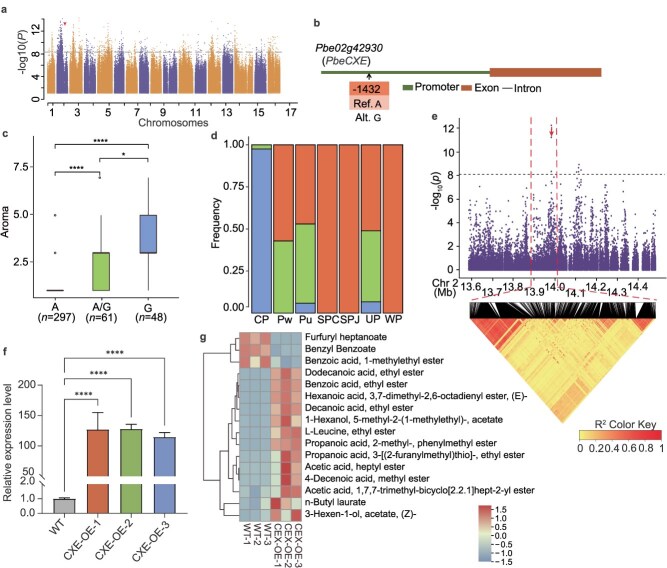
GWAS of pear fruit aroma and identification of *PbeCXE* as a candidate gene. **a**, Manhattan plot of GWAS −log_10_(*P*-values) indicating the strength of SNP association with fruit aroma. **b**, Gene structure of *Pbe02g42930* (*PbeCXE*). **c**, Box plot of fruit aroma in relation to the genotype of the SNP (Chr2_13,982 568). **d**, Variation in *Pbe02g42930* (*PbeCXE*) haplotype frequency among groups of pear accessions. **e**, Local Manhattan plot (top) and linkage disequilibrium heatmap (bottom) surrounding the peak on chromosome 2. **f**, Confirmation of stable *PbeCXE* transgene integration in ‘Clapp’s Favourite’ callus by qRT-PCR. Data are presented as the mean ± SD from three independent biological replicates. Statistical significance was determined using Student’s *t*-test (^*^  *P* < 0.05, ^**^  *P* < 0.01, ^***^  *P* < 0.001). **g**, Gas chromatography–tandem mass spectrometry analysis of volatile compound contents in ‘Clapp’s Favourite’ callus.

Aroma is a crucial sensory property that strongly contributes to the flavor quality of pear fruit. The biochemical basis of aroma lies in volatile metabolites, particularly short- to medium-chain alcohol esters, such as ethyl and methyl esters, that determine the characteristic aroma and sensory perception of the fruit [48]. Among the 291 identified aroma-associated SNPs, 101 (34.7%) were located on chromosome 2 (Fig. 6a and e). The present GWAS detected a strong signal at the locus corresponding to the carboxylesterase gene *Pbe02g42930* (*PbeCXE*). In apple, *MdCXE1* encodes a carboxylesterase that modulates the ripe fruit aroma [49]. Similarly, in strawberry, *FanCXE1* encodes a carboxylesterase involved in the catabolism of volatile esters during fruit ripening [50]. Regarding this aroma-associated volatile metabolite locus (Chr2_13,982 568; *PbeCXE* promoter) (Fig. 6b), the accessions carrying the G/G allele exhibited higher volatile metabolite accumulation compared with that of the accessions carrying the A/A or A/G allele (Fig. 6c). Almost all CP accessions with aromatic fruit harbored the G/G allele (Fig. 6d).

Metabolomic analysis of volatile metabolites revealed substantial variation in the abundance of esters, alcohols, ketones, terpenoids, and aldehydes—critical contributors to fruit aroma [51]—in European pear ‘Clapp's Favourite’ callus overexpressing *PbeCXE* compared with those of the wild type (WT) (Fig. 6f). Notably, accumulation of several pear volatile ester compounds was remarkably increased. Specifically, the contents of ethyl benzoate, heptyl acetate, methyl dec-4-enoate, and (3Z)-3-hexen-1-yl acetate were 20.45-, 12.54-, 11.57-, and 2.64-fold higher than those in the WT, respectively, contributing significantly to the formation of the pear fruit aroma [52, 53]. In contrast, the content of propan-2-yl benzoate, which is involved in carboxylesterase metabolite, decreased by 0.49-fold to 0.0079 μg/g compared with that of the WT (Fig. 6f and g, Supplementary Tables S15 and S16). Transient expression of *PbeCXE* in pear ‘Whangkeumbae’ fruit led to significant alterations in the concentrations of volatile metabolites. Notably, the contents of nonyl acetate and ethyl benzoate—aroma compounds previously identified in strawberry and pear [54, 55]—were substantially increased to 0.012 and 0.019 μg/g, representing 6.32- and 11.70-fold increases compared with those of the control, respectively. In contrast, the contents of benzyl acetate, 2-isopropenyl-5-methyl-4-hexen-1-yl acetate, 2-methylpropyl 2-phenylacetate, and 3-methylbut-2-enyl hexanoate—which are substrates for carboxylesterase enzymatic oxidation [50, 56]—were markedly reduced to 0.0093, 0.018, 0.0046, and 0.0054 μg/g, corresponding to 0.68-, 0.44-, 0.39-, and 0.58-fold decreases, respectively. This degradation plays a pivotal role in maintaining a dynamic balance of volatile metabolites, reducing the abundance of less aromatic precursors while driving the metabolic flux towards the biosynthesis of critical aroma-contributing esters. Collectively, these results suggest that *PbeCXE* plays a crucial role in modulating pear fruit aroma-associated volatile metabolites by influencing the balance between ester biosynthesis and substrate consumption, thereby enhancing the aromatic profile of the fruit (Supplementary Fig. S16a and b, Supplementary Tables S17 and S18).

Skin color is an important phenotypic trait of pear fruit (Supplementary Fig. S17a). The signal associated with this trait was mapped to the 3.9- to 5.4-Mb interval on chromosome 8 (Supplementary Fig. S17c). This region included *Pbe08g54210* (*PbeMYB38*), which was annotated as an MYB transcription factor gene. MYB transcription factors are regulators of skin color in sand pear fruit [6, 17]. The present qRT-PCR data indicated that *PbeMYB38* was more highly expressed in accessions with a russet fruit skin than in accessions with a yellow fruit skin (Supplementary Fig. S18). The diversity in this trait was strongly associated with an SNP (G/A) in *PbeMYB38* (base pair position 881) (Supplementary Fig. S17b and d). The accessions with a greenish-yellow or yellow fruit skin predominantly carried the G/G allele, whereas the accessions with a russet fruit skin primarily carried the A/A allele (Supplementary Fig. S17e and f).

## Discussion

Given the complex evolutionary history of *Pyrus*, which originated in western China [1, 2] and subsequently underwent domestication and improvement [6, 13], extant pear germplasm is derived from intraspecific and interspecific hybridization, and exhibits extensive morphological and physiological diversity. The present investigation into the dispersal patterns and genetic interactions among *Pyrus* species sheds light on the important impacts of geography and human influence on the evolutionary history of pear. We identified the Yunnan–Guizhou Plateau as a dissemination center for Asian cultivated *P. pyrifolia* and *P. bretschneideri*, emphasizing the importance of this region in pear diversity and distribution. Debate regarding the origin of Japanese pear is ongoing [13–16]. We detected gene flow from the ancestral component of Pu to SPJ, which implied the possible dispersal of wild *Pyrus* species from northeastern mainland China to the Japanese archipelago, probably along the Korean Peninsula and across a considerably narrower Tsushima Strait due to lowering of the sea level during glacial periods [57].

We have documented genetic introgression among pear populations, demonstrating the intricate genetic interactions that have shaped pear germplasm, influenced by both natural hybridization and agricultural practices. In Northwest China, the geographical distribution of these genetic interactions is exemplified by two genetically admixed populations, NWC-1 and NWC-2, which exhibit differing degrees of admixture between European pear and Asian pear. This result is consistent with previous findings that highlight *P. sinkiangensis* as an example of such hybridization [6]. In Northeast China, we detected strong gene flow from Pu to UP, and introgression between UP and WP. These findings support the contention that cultivated *P. ussuriensis* was originally domesticated from wild *P. ussuriensis* [6], with subsequent hybridization between wild *P. ussuriensis* and *P. bretschneideri*, or between cultivated *P. ussuriensis* and *P. bretschneideri*, especially for certain commercially important UP cultivars [13]. The taxonomy of WP and its relationship with SP remains ambiguous, primarily because of the lack of a wild ancestor [13, 14]. The present genomic analysis suggests that a re-evaluation of the classification of pear varieties is warranted. Specifically, pear accessions in North China and the West Sichuan Plateau traditionally recognized as *P. bretschneideri* [3, 4] based on phenotypic traits are aligned more closely on a genomic level with *P. pyrifolia*.

The domestication of pears exerted only a modest bottleneck effect, with only a slight reduction in genetic diversity, whereas subsequent improvement efforts have typically resulted in more substantial losses of diversity [58]. Despite this, breeding initiatives have notably enhanced genetic exchange (Fig. 2e) and diversity within hybrid populations (Fig. 2a), especially in groups such as UP, NWC-1, and NWC-2. The high nucleotide diversity in NWC-1 and NWC-2 is not solely attributable to broad geographic sampling but also reflects genuine admixture shaped by the domestication and improvement history of pear. Modern cultivars frequently harbor contributions from two major genetic pools—one largely eastern, the other largely western—due to historical germplasm exchange and hybrid breeding. NWC-1 and NWC-2, although genetically distant from each other, exemplify parallel outcomes of these processes, supporting the opinion that such mixture is a hallmark of pear improvement.

This significant enhancement likely bestows upon these groups improved performance capabilities and greater adaptive potential. Similarly, in citrus (*Citrus* spp.), hybridization and breeding efforts have not only preserved, but in some cases even increased, genetic diversity within certain cultivated varieties [59]. The constructive role of hybridization in augmenting the genetic repertoire for pear improvement underscores its contribution to enhancing biodiversity among cultivated pears.

In the MSMC analysis, we observed distinct population dynamics among *Pyrus* species, gaining insights into their evolutionary trajectories in response to climatic events. Most cultivated species, including *P. communis*, *P. bretschneideri*, and *P. pyrifoli*a, experienced significant population bottlenecks during the LGM, suggesting their vulnerability to the harsh climatic conditions. Conversely, wild *P. betulifolia* demonstrated greater resilience, showing only a modest decline in genetic diversity during the LGM. This finding indicates *P. betulifolia* shows robust adaptive potential, highlighting the importance of preserving wild germplasm to enhance abiotic stress tolerance in modern breeding.

The GWAS and genome-wide selective-sweep analysis identified multiple critical agronomic traits. Notably, *WUS* and *CLV* were consistently selected in all comparisons between wild and cultivated pear, whereas genes, such as *CKI*, *CYCD*, *E3RNF*, and *YABBY*, were exclusively selected in comparisons involving Asian pear. These findings accord with previous studies that highlighted distinct genomic regions subject to selection in Asian pear and European pear [6]. Furthermore, acidity and astringency are traits associated with domestication, and both the GWAS and genome-wide selection analysis indicated that *PIN3* and *PPO* contribute to pear fruit acidity and astringency, respectively.

Recent studies have identified significant SNPs associated with alcohol acyltransferase (AAT), a critical enzyme in ester biosynthesis, in apple and peach [60, 61]. In the present analysis, we detected a highly significant A/G SNP mutation in *PbeCXE*, which is a key gene involved in ester metabolism. This study establishes the association between SNPs and the *AAT* and *CXE* genes in regulating ester biosynthesis and degradation, thereby influencing fruit aroma. These findings highlight molecular targets for improving fruit aroma through breeding and metabolic engineering. In addition, we identified a novel nonsynonymous SNP within the conserved domain of *PbePPO*, which may affect the accumulation of polyphenols associated with astringency [62]. Notably, astringent pear accessions predominantly carried the G/G allele, which is consistent with the findings from apple [63]. Our results provide novel insights into the genetics of agronomic traits and the evolutionary history of pear. To further validate our hypotheses, more systematic collection and analysis of morphological descriptors should be incorporated in future studies.

## Conclusion

This study provides insights into the evolutionary history and domestication of pear based on a comprehensive genomic analysis. The Yunnan–Guizhou Plateau is identified as a critical dissemination center for Asian cultivated pears and extensive interspecific introgression is revealed. The genomic footprints of gene flow indicate complex domestication pathways, contributing to improved knowledge of the ancestry of extant germplasm. The identified genes (e.g. *PbeMADS25*, *PbeDHQ-SDH*, *PbeCXE*, *PbePIN3*, and *PbePPO*) underlying important agronomic traits are potential molecular targets for improvement of fruit quality and will facilitate the use of precision molecular breeding approaches to generate novel cultivars with enhanced flavor profiles. In addition to the pear-specific insights, the present findings establish a genomic framework for conducting evolutionary and domestication analyses of perennial Rosaceae fruit crops characterized by complex hybridization histories.

## Materials and Methods

**Plant materials and phenotyping**. The 495 pear accessions analyzed in this study comprised the following: 67 *P. bretschneideri*, 139 Chinese *P. pyrifolia*, 34 Japanese and Korean *P. pyrifolia*, 47 cultivated *P. ussuriensis*, 19 wild *P. ussuriensis*, 20 *P. sinkiangensis*, 42 *P. communis*, 4 *P. calleryana*, 13 *P. betulifolia*, 1 *P. hopeiensis*, 13 *P. phaeocarpa*, 2 *P. serrulata*, 6 *P. xerophila*, 12 unknown origin, and 76 Chinese hybrid-selected cultivars preserved in the Chinese National Germplasm Repository of Pear, Research Institute of Pomology, Chinese Academy of Agricultural Sciences (Supplementary Fig. S1, Supplementary Tables S1 and S14).

The phenotypic traits were categorized either as fruit characteristics (astringency, aroma, acidity, and skin color) or plant architecture (number of stigmas, number of locules, number of stamens, young leaf color, and sepal persistence). The traits were assessed in accordance with *Description and Data Standard for Pear* (*Pyrus spp*.) [18].

The aroma intensity of the fruit was evaluated based on a standardized sensory test with reference to international guidelines [64]. To ensure the reliability of the evaluation, the testing environment was odor-free, and evaluators were confirmed to have normal olfactory function without medical conditions, such as colds, that might impair their sense of smell. A panel of five to eight evaluators with normal olfactory sensitivity was selected. Each evaluator assessed the aroma of 10 fruit flesh samples. For crisp-fleshed accessions, the fruit were harvested at full maturity and evaluated immediately after harvest. For soft-fleshed accessions (including European pears and *P. ussuriensis*), the fruit were harvested at full maturity, then stored until the flesh had softened through ripening before evaluation. The evaluators rated the aroma intensity on a four-point scale: 1, aroma absent or mostly absent; 3, weakly aromatic; 5, aromatic; and 7, strongly aromatic. After the evaluation, the ratings from all evaluators were compiled and analyzed. The average score was calculated to derive the aroma intensity of each sample.

**DNA extraction and sequencing**. Genomic DNA was extracted from fresh leaves using the cetyltrimethylammonium bromide method [65]. For each accession, at least 6 μg genomic DNA and the TruSeq Nano DNA Sample Preparation Kit (Illumina) were used to construct paired-end sequencing libraries, which were sequenced using the NovaSeq 6000 platform (150PE) by Berry Genomics Co. Ltd., Beijing, China.

**SNP detection and annotation**. The paired-end reads were first mapped to *Pbe-SD* using the Burrows–Wheeler aligner software [BWA mem (version 0.7.12)] [66]. Duplicated reads were filtered using MarkDuplicates in Picard (version 1.57; https://broadinstitute.github.io/picard/). The HaplotypeCaller module in GATK (version 4.0.3.0) [67] was used for the local realignment performed to enhance the alignments. The gvcf file of each sample was generated and then merged into one vcf file using two GATK subcomponents (CombineGVCFs and GenotypeGVCFs). To decrease the variant false discovery rate, the sites identified by GATK were filtered using the SelectVariants and VariantFiltration packages and then VCFtools (version 0.1.13) [68]. The parameters were as follows: (1) QD < 2.0 || MQ < 40.0 || MQRankSum < −12.5 || ReadPosRankSum < −8.0 applied to SNPs, and QD < 2.0 || FS > 200.0; (2)—maf 0.05—min-alleles 2—max-alleles 2—max-missing 0.95. All identified SNPs that were screened for quality were further annotated in accordance with the reference genome using ANNOVAR [69]. The SNPs were then localized to exons, splicing sites, 5′ and 3′ untranslated regions, introns, upstream and downstream regions, and intergenic regions.

**Genome-wide association study**. We selected 11 031 864 SNPs (minor allele frequency > 0.05 and missing rate < 95%) to perform GWAS for all traits. The GWAS was conducted with a linear mixed model that was implemented in FaST-LMM (version 0.4.1) [70]. A kinship matrix and the first three principal components were included as random effects. The modified Bonferroni correction method was used to determine the genome-wide significance thresholds on the basis of a nominal level of α = 0.05 [71] and the corresponding raw *P*-value of 4.53 × 10^−9^.

**qRT-PCR analysis**. Total RNA was extracted using the TRNzol Universal Reagent (TIANGEN, Beijing, China). The cDNA was synthesized with the cDNA Synthesis Kit (Takara, Dalian, China). Candidate gene sequences were obtained from the Genome Database for Rosaceae (GDR; https://www.rosaceae.org). The qRT-PCR primers used are listed in Supplementary Table S13. The qRT-PCR reactions were conducted on a LightCycler 96 System (Roche Diagnostics) in accordance with the manufacturer’s instructions. Relative gene expression levels were calculated using the 2^−ΔΔ*C*q^ method [72].

**Generation and identification of calli overexpressing**  *PbeCXE*. European pear ‘Clapp’s Favourite’ calli were immersed for 20 min at 25°C in an *Agrobacterium tumefaciens* cell suspension harboring the *PbeCXE* overexpression constructs in the pK7WG2D vector. The bacterial suspension was prepared in liquid infection medium supplemented with 100 μM acetosyringone. Following infection, the calli were cocultured on solid Murashige and Skoog (MS) medium supplemented with 0.5 mg·L^−1^ 6-benzylaminopurine (6-BA) and 1 mg·L^−1^ 2, 4-dichlorophenoxyacetic acid (2,4-D) at 25°C for 2 d in the dark, then cultured on solid MS medium supplemented with 0.5 mg·L^−1^ 6-BA, 1 mg·L^−1^ 2, 4-D, and 20 mg·L^−1^ hygromycin for at least 2 months at 25°C in the dark. Transgenic calli were subcultured every 21 d for a total of three cycles. Putative transgenic calli were identified based on fluorescence analysis and confirmed by qRT-PCR using gene-specific primers.

**Population genetics analysis**. We used the whole-genome SNPs to construct a maximum-likelihood phylogenetic tree with 100 bootstrap replicates using RAxML (version 8.2.12) [73]. *Malus pumila* was included as the outgroup. iTOL (http://itol.embl.de) was used to visualize the phylogenetic tree. The SNPs in LD were filtered using PLINK (version 1.90b3.38) [74], with a window size of 50 SNPs and an *r*^2^ threshold of 0.2. The PCA was performed using the Genome-wide Complex Trait Analysis software (version 1.25.3) [75] and the first three eigenvectors were plotted. The population structure was analyzed using the block-relaxation algorithm implemented in ADMIXTURE (version 1.3) [76]. To explore the convergence of individuals, we predefined the number of genetic clusters *K* from 2 to 13 and calculated the cross-validation error. Default methods and settings were used in the analyses. In addition, LD was calculated using PopLDdecay (version v3.31) [77]. The pairwise *r*^2^ values within and between different chromosomes were calculated. The LD for each group was calculated only using the SNP pairs from the corresponding group.

**Demographic history reconstruction using MSMC**. We used the MSMC [78] model to infer population size (*N*_e_). The input files were generated using MSMC Tools (https://github.com/stschiff/msmc-tools). A generation time of 5 years and a mutation rate of 3.5 × 10^−9^ mutations per nucleotide per year were used to convert the scaled times and population sizes into real times and sizes.

**Genome scanning for selective-sweep signals**. The *F*_st_ and nucleotide diversity (*π*) were calculated using VCFtools (version 0.1.16) and pixy [79] with 10 kb sliding windows and 5 kb steps. The domestication regions (Pw vs. other groups) were defined on the basis of the weighted *F*_st_ value and the ratio of the *π* value in each composition window (cutoff: top 5%).

The XP-CLR method [80] was used to screen for genome-wide selective sweeps. Briefly, we screened for selective sweeps using 10 kb windows. The top 10% of XP-CLR scores were used to select the putative domestication regions.

**Detection of gene flow**. Population relatedness and dispersal events were inferred using TreeMix [81]. The optimal number of migration edges was calculated with OptM [82]. The tree was constructed using CP as the outgroup. *D*-statistics [83] were calculated within a nonoverlapping 100 kb window to detect asymmetric gene flow within a specified four-taxon tree ‘((P1,P2),P3),O’.

**Estimation of mutation rate**. The pear mutation rate was calculated using the formula μ = *D*/2 *T*, where *D* is the evolutionary distance of pear and apple and *T* is the divergence time of the two species (500 Mya). OrthoFinder [84] was used to define single-copy orthologous genes in the two species. Multiple single-copy genes were aligned using Muscle. The mutation rate was estimated to be 3.5 × 10^−9^ substitutions per site per year.

**Measurement of malic acid and aroma metabolites in the fruit**. The target gene was cloned into the Gateway vector pK7WG2D and subsequently transformed into *A. tumefaciens* strain GV3101 for *in vivo* injection. Three experimental groups were established: a control group, an empty Gateway vector group, and an overexpression vector group for the target gene. Thirty ‘Whangkeumbae’ fruit were injected per group and samples from the injection sites were collected at 3 d post-injection. The malic acid content was analyzed using high-performance liquid chromatography (e2695, Waters, USA). Aroma metabolites were quantified using gas chromatography–tandem mass spectrometry (8890-7000D, Agilent, USA).

## Supplementary Material

Web_Material_uhag042

## Data Availability

The short-read genomic resequencing data for 495 *Pyrus* accessions generated in this study have been deposited with the NCBI under BioProject ID PRJNA1102892.

## References

[1] Bailey LH . Pyrus stand cyclopedia Hort. 1917;5:2865–78

[2] Rubtsov GA . Geographical distribution of the genus *Pyrus* and trends and factors in its evolution. Am Nat. 1944;78:358–66

[3] Pu FS, Wang YL. Pomology of China: Pears. Shanghai, China: Shanghai Scientific and Technical Publishers, 1963

[4] Cao YF, Zhang SL. Pear genetic resource in China. Beijing, China: China Agriculture Press, 2020

[5] Challice JS, Westwood MN. Numerical taxonomic studies of the genus *Pyrus* using both chemical and botanical characters. Bot J Linn Soc. 1973;67:121–48

[6] Wu J, Wang YT, Xu JB. et al. Diversification and independent domestication of Asian and European pears. Genome Biol. 2018;19:77 29890997 10.1186/s13059-018-1452-y PMC5996476

[7] Li JM, Wang Y, Zhang SL. et al. Pear genetics: recent advances, new prospects, and a roadmap for the future. Hortic Res. 2022;9:uhab040 35031796 10.1093/hr/uhab040 PMC8778596

[8] Jin ZT, Lin XH, Ma DK. et al. Unravelling the web of life: incomplete lineage sorting and hybridisation as primary mechanisms over polyploidisation in the evolutionary dynamics of pear species. Mol Ecol Resour. 2025;25:e70029 40797299 10.1111/1755-0998.70029

[9] Müller JV . Domestication of wild pears in Europe, with specific emphasis on the Caucasian endemic pear *Pyrus communis* subsp. *caucasica* (Fed.) Browicz. Hortic Sci Biotechnol. 2024;99:1–8

[10] Vidaković A, Šatović Z, Liber Z. et al. Genetic diversity of *Pyrus pyraster* (L.) Burgsd. and *P. spinosa* forssk.: evidence of introgression from cultivated into wild pear populations. Trees. 2024;38:1297–314

[11] Xue L, Liu Q, Hu H. et al. The southwestern origin and eastward dispersal of pear (*Pyrus pyrifolia*) in East Asia revealed by comprehensive genetic structure analysis with SSR markers. Tree Genet Genomes. 2018;14:48

[12] Chen XN, Li JM, Zhang SL. Genome-wide genetic diversity and IBD analysis reveals historic dissemination routes of pear in China. Tree Genet Genomes. 2022;18:1

[13] Yue XY, Zheng XC, Li JM. et al. Combined analyses of chloroplast DNA haplotypes and microsatellite markers reveal new insights into the origin and dissemination route of cultivated pears native to East Asia. Front Plant Sci. 2018;9:591 29868056 10.3389/fpls.2018.00591 PMC5949605

[14] Rehder A . Manual of Cultivated Trees and Shrubs (2^nd^ Ed). London, UK: Macmillan, 1940

[15] Kikuchi A . Assessment of China pear species and cultivars. Collect Rec Hort Res Fac Agri Kyoto Univ. 1946;3:1–11

[16] Kikuchi A . Horticulture of Fruit Trees. Tokyo, Japan: Yokendo, 1948

[17] Zhang MY, Xue C, Hu H. et al. Genome-wide association studies provide insights into the genetic determination of fruit traits of pear. Nat Commun. 2021;12:1144 33602909 10.1038/s41467-021-21378-y PMC7892570

[18] Cao YF, Liu FZ, Hu HJ. et al. Descriptors and data standard for pear (*Pyrus* spp.). Beijing, China: China Agriculture Press, 2006

[19] Zhang Y, Cao YF, Huo HL. et al. Diversity of pear germplasm resources based on twig and leaf phenotypic traits. Sci Agric Sin. 2018;51:3353–69

[20] Yamamoto T, Terakami S, Takada N. et al. Identification of QTLs controlling harvest time and fruit skin color in Japanese pear (*Pyrus pyrifolia* Nakai). Breed Sci. 2014;64:351–61 25914590 10.1270/jsbbs.64.351 PMC4267310

[21] Wu J, Li LT, Li M. et al. High-density genetic linkage map construction and identification of fruit-related QTLs in pear using SNP and SSR markers. J Exp Bot. 2014;65:5771–81 25129128 10.1093/jxb/eru311 PMC4203118

[22] Iwata H, Hayashi T, Terakami S. et al. Potential assessment of genome-wide association study and genomic selection in Japanese pear *Pyrus pyrifolia*. Breed Sci. 2013;63:125–40 23641189 10.1270/jsbbs.63.125 PMC3621438

[23] Dong XG, Wang Z, Tian LM. et al. De novo assembly of a wild pear (*Pyrus betuleafolia*) genome. Plant Biotechnol J. 2020;18:581–95 31368610 10.1111/pbi.13226 PMC6953202

[24] Wu J, Wang ZW, Shi ZB. et al. The genome of the pear (*Pyrus bretschneideri* Rehd). Genome Res. 2013;23:396–408 23149293 10.1101/gr.144311.112 PMC3561880

[25] Jing DL, Liu XY, He Q. et al. Genome assembly of wild loquat (*Eriobotrya japonica*) and resequencing provide new insights into the genomic evolution and fruit domestication in loquat. Hortic Res. 2023;10:uhac265 36778182 10.1093/hr/uhac265 PMC9909508

[26] Huang ZY, Shen F, Chen Y. et al. Chromosome-scale genome assembly and population genomics provide insights into the adaptation, domestication, and flavonoid metabolism of Chinese plum. Plant J. 2021;108:1174–92 34473873 10.1111/tpj.15482

[27] Xu JJ, Fang X, Li CY. et al. General and specialized tyrosine metabolism pathways in plants. Abiotech. 2020;1:97–105 36304719 10.1007/s42994-019-00006-w PMC9590561

[28] Luo DL, Wang XG, Chen JY. et al. Analysis of α-linolenic acid metabolism in ‘Fengtang’ plum across different maturity stages. Sci Hortic. 2025;346:114173

[29] Pang LL, Wu Y, Pan YF. et al. Insights into exogenous melatonin associated with phenylalanine metabolism in postharvest strawberry. Postharvest Biol Technol. 2020;168:111244

[30] Harada T, Torii Y, Morita S. et al. Cloning, characterization, and expression of xyloglucan endotransglucosylase/hydrolase and expansin genes associated with petal growth and development during carnation flower opening. J Exp Bot. 2011;62:815–23 20959626 10.1093/jxb/erq319 PMC3003822

[31] Groppi A, Liu S, Cornille A. et al. Population genomics of apricots unravels domestication history and adaptive events. Nat Commun. 2021;12:3956 34172741 10.1038/s41467-021-24283-6 PMC8233370

[32] Fang C, Ma YM, Wu SW. et al. Genome-wide association studies dissect the genetic networks underlying agronomical traits in soybean. Genome Biol. 2017;18:161 28838319 10.1186/s13059-017-1289-9 PMC5571659

[33] Rodríguez-Leal D, Lemmon ZH, Man J. et al. Engineering quantitative trait variation for crop improvement by genome editing. Cell. 2017;171:470–80 28919077 10.1016/j.cell.2017.08.030

[34] Cheng X, Xiong Y, Li DH. et al. Bioinformatic and expression analysis of the OMT gene family in *Pyrus bretschneideri* cv. Dangshan Su. Genet Mol Res. 2016;15: gmr.15038664 10.4238/gmr.15038664 27706700

[35] Martínez-Rivas FJ, Blanco-Portales R, Moyano E. et al. Strawberry fruit FanCXE1 carboxylesterase is involved in the catabolism of volatile esters during the ripening process. Hortic Res. 2022;9:uhac095 35795396 10.1093/hr/uhac095 PMC9249579

[36] Wu ZJ, Liang GD, Li YY. et al. Transcriptome and metabolome analyses provide insights into the composition and biosynthesis of grassy aroma volatiles in white-fleshed pitaya. ACS Omega. 2022;7:6518–30 35252648 10.1021/acsomega.1c05340 PMC8892475

[37] Li YZ, He LL, Song YH. et al. Comprehensive study of volatile compounds and transcriptome data providing genes for grape aroma. BMC Plant Biol. 2023;23:171 37003985 10.1186/s12870-023-04191-1 PMC10064686

[38] Zhang Y, Cao YF, Huo HL. et al. An assessment of the genetic diversity of pear (*Pyrus* L.) germplasm resources based on the fruit phenotypic traits. J Integr Agric. 2022;21:2275–90

[39] Purugganan MD, Rounsley SD, Schmidt RJ. et al. Molecular evolution of flower development: diversification of the plant MADS-box regulatory gene family. Genetics. 1995;140:345–56 7635298 10.1093/genetics/140.1.345 PMC1206560

[40] Han SW, Laura G, Danny JS. The signal peptide peptidase is required for pollen function in *Arabidopsis*. Plant Physiol. 2009;149:1289–301 19168645 10.1104/pp.108.130252 PMC2649412

[41] Zhang Y, Cao YF, Huo HL. et al. Research on diversity of pear germplasm resources based on flowers phenotype traits. Acta Hortic Sin. 2016;43:1245–56

[42] Jiang B, Chen LJ, Duan SM. et al. Transcriptomic analysis identifies differentially expressed genes in purple tender shoots and green mature leaves of Zijuan tea. Czech J Food Sci. 2022;40:210–20

[43] Liu YL, Zhang XJ, Zhao ZY. Effects of fruit bagging on anthocyanins, sugars, organic acids, and color properties of‘Granny Smith’and‘Golden Delicious’during fruit maturation. Eur Food Res Technol. 2013;236:329–39

[44] Ellis CM, Nagpal P, Young JC. et al. Auxin response factor 1 and auxin response factor 2 regulate senescence and floral organ abscission in *Arabidopsis thaliana*. Development. 2005;132:4563–74 16176952 10.1242/dev.02012

[45] He M, Tian HL, Luo XW. et al. Molecular progress in research on fruit astringency. Molecules. 2015;20:1434–51 25599149 10.3390/molecules20011434 PMC6272358

[46] Guo XY, Lv YQ, Ye Y. et al. Polyphenol oxidase dominates the conversions of flavonol glycosides in tea leaves. Food Chem. 2021;339:128088 32979714 10.1016/j.foodchem.2020.128088

[47] Cao K, Zhou ZK, Wang Q. et al. Genome-wide association study of 12 agronomic traits in peach. Nat Commun. 2016;7:13246 27824331 10.1038/ncomms13246 PMC5105138

[48] Suwanagul A, Richardson G. Identification of headspace volatile compounds from different pear (*Pyrus communis* L.) varieties. Acta Hortic. 1998;475:605–24

[49] Souleyre E, Marshall S, Oakeshott J. et al. Biochemical characterisation of MdCXE1, a carboxylesterase from apple that is expressed during fruit ripening. Phytochemistry. 2011;72:564–71 21315388 10.1016/j.phytochem.2011.01.020

[50] Félix JMR, Rosario BP, Enriqueta M. et al. Strawberry fruit FanCXE1 carboxylesterase is involved in the catabolism of volatile esters during the ripening process. Hortic Res. 2022;9:uhac095 35795396 10.1093/hr/uhac095 PMC9249579

[51] Wang XH, Chen YY, Zhang JJ. et al. Comparative analysis of volatile aromatic compounds from a wide range of pear (*Pyrus* L.) germplasm resources based on HS-SPME with GC–MS. Food Chem. 2023;418:135963 36944308 10.1016/j.foodchem.2023.135963

[52] Zhang ZM, Zeng DD, Li GK. The study of the aroma profile characteristics of durianpulp during storage by the combination sampling method coupled with GC–MS. Flavour Fragr J. 2007;22:71–7

[53] Chen JQ, Lü JH, He ZS. et al. Investigations into the production of volatile compounds in Korla fragrant pears (*Pyrus sinkiangensis* Yu). Food Chem. 2020;302:125337 31419770 10.1016/j.foodchem.2019.125337

[54] Zhang LJ, Zhou K, Wang MH. et al. The functional characterization of carboxylesterases involved in the degradation of volatile esters produced in strawberry fruits. Int J Mol Sci. 2022;24:383 36613824 10.3390/ijms24010383 PMC9820763

[55] Versini G, Franco MA, Moser S. et al. Characterisation of pear distillates from wild and cultivated varieties in Sardinia. Int J Food Sci Technol. 2012;47:2519–31

[56] Qi LY, Li XJ, Zang NN. et al. Genome-wide identification of CXE and PuCXE15 functions in the catabolism of volatile ester in ‘Nanguo’ pear fruit. Plant Physiol Biochem. 2023;203:107996 37688900 10.1016/j.plaphy.2023.107996

[57] Oba T, Kato M, Kitazato H. et al. Paleoenvironmental changes in the Japan Sea during the last 85,000 years. Paleoceanography. 1991;6:499–518

[58] Li JM, Zhang MY, Li XL. et al. Pear genetics: recent advances, new prospects, and a roadmap for the future. Hortic Res. 2022;9:uhab040 35031796 10.1093/hr/uhab040 PMC8778596

[59] Wu GA, Prochnik S, Jenkins J. et al. Sequencing of diverse mandarin, pummelo and orange genomes reveals complex history of admixture during citrus domestication. NatBiotechnol. 2014;32:656–62 10.1038/nbt.2906 PMC4113729 24908277

[60] Soomro T, Jordan M, Watts S. et al. Genomic insights into apple aroma diversity. Fruit Res. 2023;3:27

[61] Cao XM, Su YK, Zhao T. et al. Multi-omics analysis unravels chemical roadmap and genetic basis for peach fruit aroma improvement. Cell Rep. 2024;43:114623 39146179 10.1016/j.celrep.2024.114623

[62] He WJ, Laaksonen O, Tian Y. et al. Chemical composition of juices made from cultivars and breeding selections of European pear (*Pyrus communis* L.). J Agric Food Chem. 2022;70:5137–50 35426665 10.1021/acs.jafc.2c00071 PMC9052750

[63] Kumar S, Deng CH, Molloy C. et al. Extreme-phenotype GWAS unravels a complex nexus between apple (*Malus domestica*) red-flesh colour and internal flesh browning. Fruit Res. 2022;2:1–14

[64] Nagamine T . The descriptors for characterization and evaluation in plant genetic resource. National Inst Agrobiological Resources of Jap. 1999;1:1–406

[65] Murray MG, Thompson WF. Rapid isolation of high molecular weight plant DNA. Nucleic Acids Res. 1980;8:4321–5 7433111 10.1093/nar/8.19.4321 PMC324241

[66] Li H, Durbin R. Fast and accurate short read alignment with Burrows–Wheeler transform. Bioinformatics. 2009;25:1754–60 19451168 10.1093/bioinformatics/btp324 PMC2705234

[67] McKenna A, Hanna M, Banks E. et al. The genome analysis toolkit: a MapReduce framework for analyzing next-generation DNA sequencing data. Genome Res. 2010;20:1297–303 20644199 10.1101/gr.107524.110 PMC2928508

[68] Danecek P, Auton A, Abecasis G. et al. The variant call format and VCFtools. Bioinformatics. 2011;27:2156–8 21653522 10.1093/bioinformatics/btr330 PMC3137218

[69] Wang K, Li MY, Hakonarson H. Annovar: functional annotation of genetic variants from high-throughput sequencing data. Nucleic Acids Res. 2010;38:e164 20601685 10.1093/nar/gkq603 PMC2938201

[70] Lippert C, Listgarten J, Liu Y. et al. FaST linear mixed models for genome-wide association studies. Nat Methods. 2011;8:833–5 21892150 10.1038/nmeth.1681

[71] Li MX, Yeung JMY, Cherny SS. et al. Evaluating the effective numbers of independent tests and significant p-value thresholds in commercial genotyping arrays and public imputation reference datasets. Hum Genet. 2012;131:747–56 22143225 10.1007/s00439-011-1118-2 PMC3325408

[72] Pfaffl MW . A new mathematical model for relative quantification in real-time RT-PCR. Nucleic Acids Res. 2001;29:e45 11328886 10.1093/nar/29.9.e45 PMC55695

[73] Stamatakis A . RAxML version 8: a tool for phylogenetic analysis and post-analysis of large phylogenies. Bioinformatics. 2014;30:1312–3 24451623 10.1093/bioinformatics/btu033 PMC3998144

[74] Purcell S, Neale B, Todd-Brown K. et al. PLINK: a tool set for whole-genome association and population-based linkage analyses. Am J Hum Genet. 2007;81:559–75 17701901 10.1086/519795 PMC1950838

[75] Yang J, Lee SH, Goddard ME. et al. GCTA: a tool for genome-wide complex trait analysis. Am J Hum Genet. 2011;88:76–82 21167468 10.1016/j.ajhg.2010.11.011 PMC3014363

[76] Alexander DH, Novembre J, Lange K. Fast model-based estimation of ancestry in unrelated individuals. Genome Res. 2009;19:1655–64 19648217 10.1101/gr.094052.109 PMC2752134

[77] Zhang C, Dong SS, Xu JY. et al. PopLDdecay: a fast and effective tool for linkage disequilibrium decay analysis based on variant call format files. Bioinformatics. 2019;35:1786–8 30321304 10.1093/bioinformatics/bty875

[78] Schiffels S, Durbin R. Inferring human population size and separation history from multiple genome sequences. Nat Genet. 2014;46:919–25 24952747 10.1038/ng.3015 PMC4116295

[79] Korunes KL, Samuk K. Pixy: unbiased estimation of nucleotide diversity and divergence in the presence of missing data. Mol Ecol Resour. 2021;21:1359–68 33453139 10.1111/1755-0998.13326 PMC8044049

[80] Chen H, Patterson N, Reich D. Population differentiation as a test for selective sweeps. Genome Res. 2010;20:393–402 20086244 10.1101/gr.100545.109 PMC2840981

[81] Pickrell J, Pritchard J. Inference of population splits and mixtures from genome-wide allele frequency data. Nat Preced. 2012;1:1 10.1371/journal.pgen.1002967 PMC3499260 23166502

[82] Fitak RR . OptM: estimating the optimal number of migration edges on population trees using TreeMix. Biol Methods Protoc. 2021;6:bpab017 34595352 10.1093/biomethods/bpab017 PMC8476930

[83] Durand EY, Patterson N, Reich D. et al. Testing for ancient admixture between closely related populations. Mol Biol Evol. 2011;28:2239–52 21325092 10.1093/molbev/msr048 PMC3144383

[84] Emms DM, Kelly S. OrthoFinder: phylogenetic orthology inference for comparative genomics. Genome Biol. 2019;20:238 31727128 10.1186/s13059-019-1832-y PMC6857279

