# Supplementary figures and images for: Whole-genome resequencing of 495 Pyrus accessions provides insights into the genetics of agronomic traits and evolutionary history of pear
Source: Hortic Res. 2026 Mar 2;13(5):uhag042. doi: 10.1093/hr/uhag042 (PMC13156035; doi:10.1093/hr/uhag042)

**a**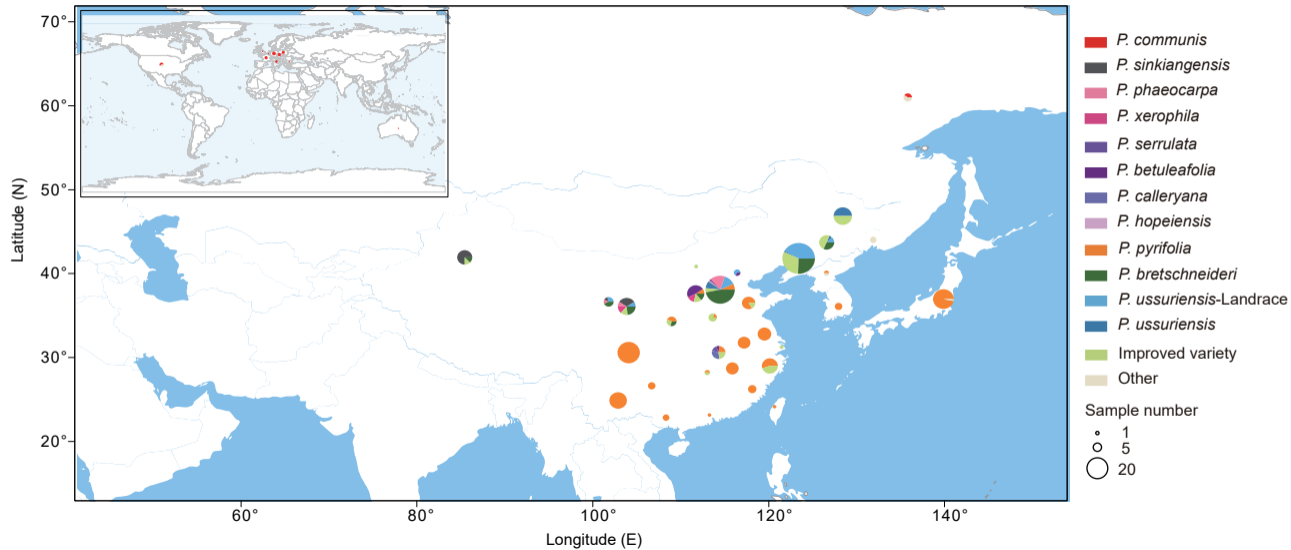

Supplement: Web_Material_uhag042 [file web_material_uhag042.zip › Supplementary Fig. 1.pdf]

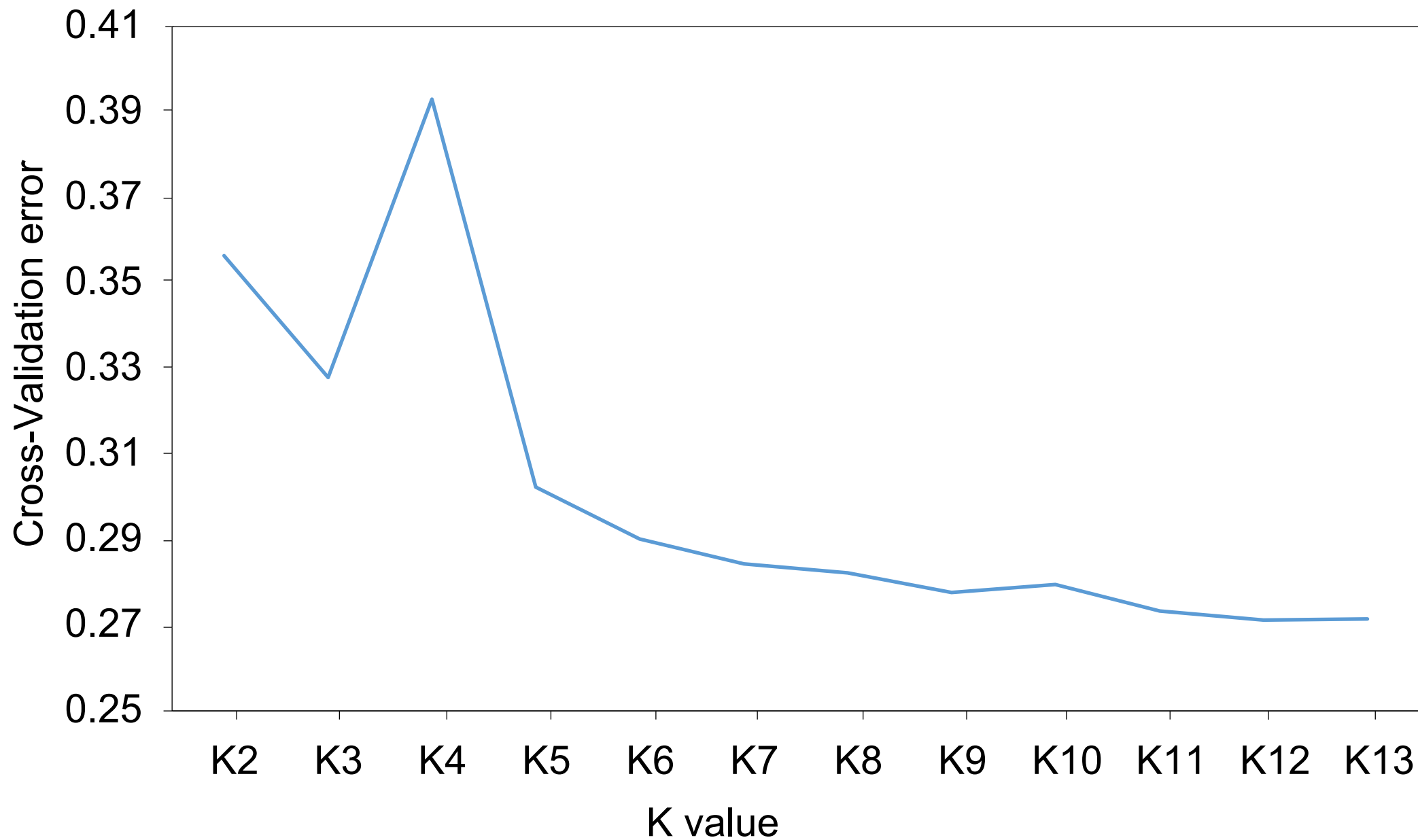

Supplement: Web_Material_uhag042 [file web_material_uhag042.zip › Supplementary Fig. 2.pdf]

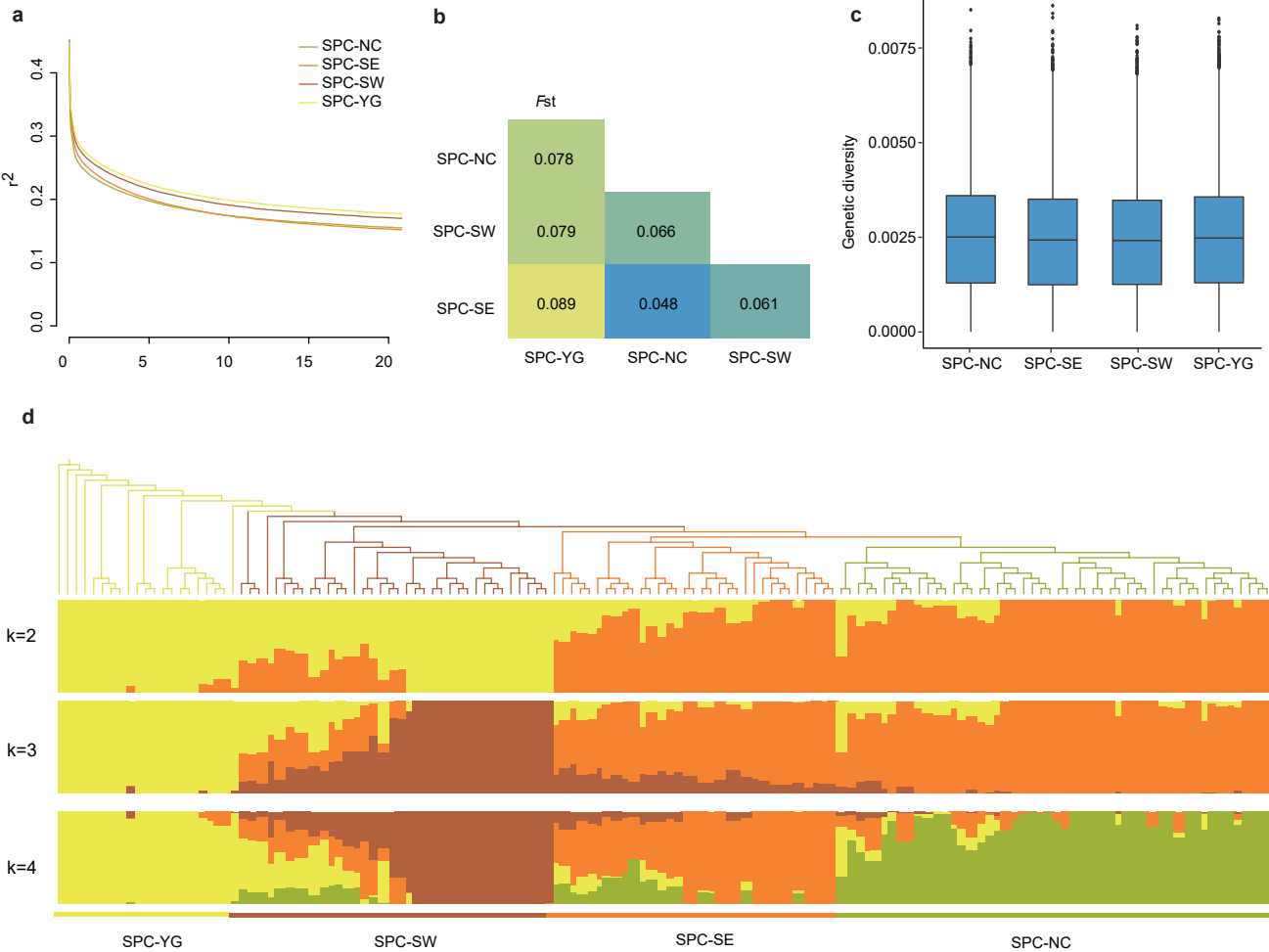

Supplement: Web_Material_uhag042 [file web_material_uhag042.zip › Supplementary Fig. 3.pdf]

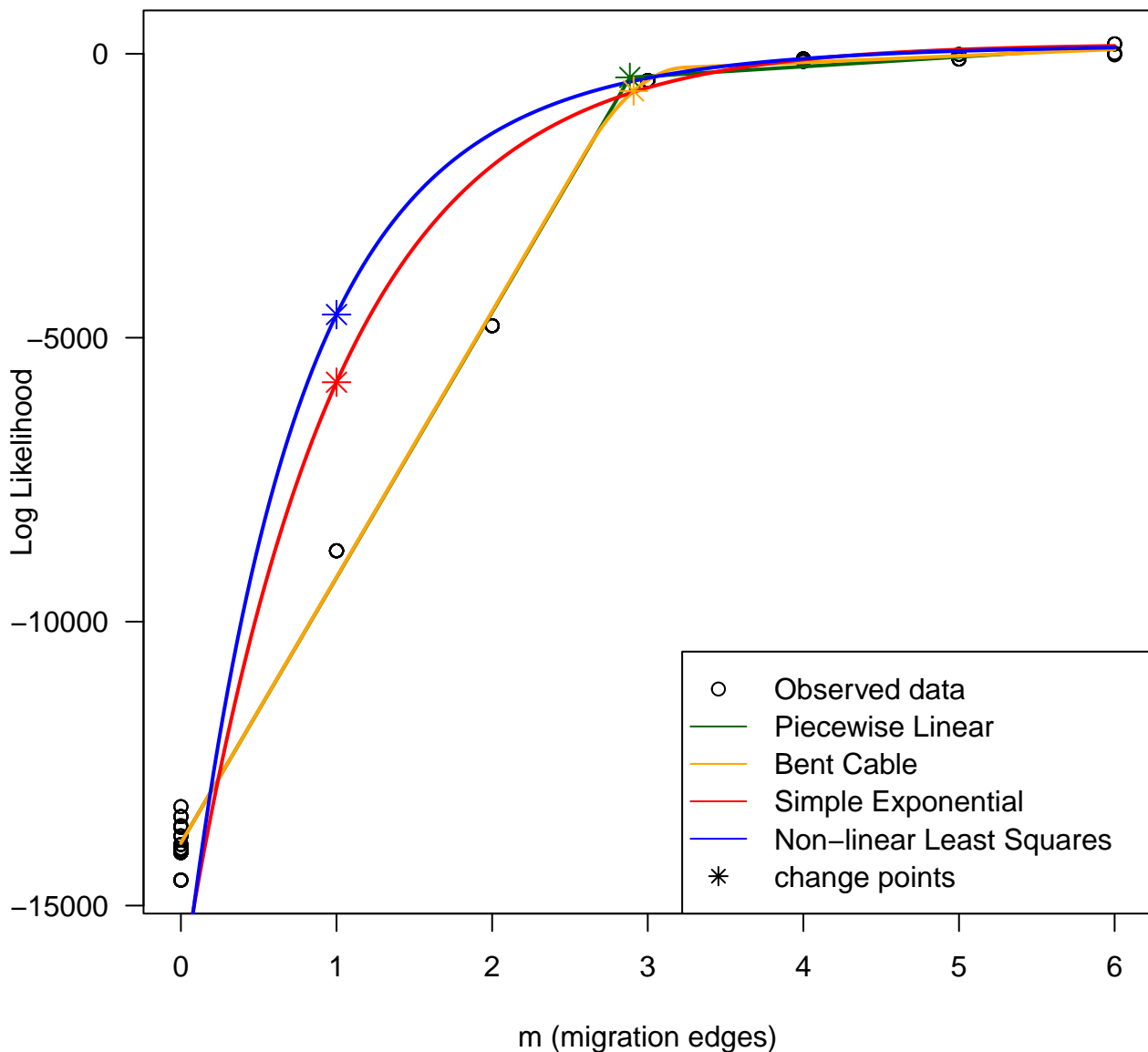

Supplement: Web_Material_uhag042 [file web_material_uhag042.zip › Supplementary Fig. 4.pdf]

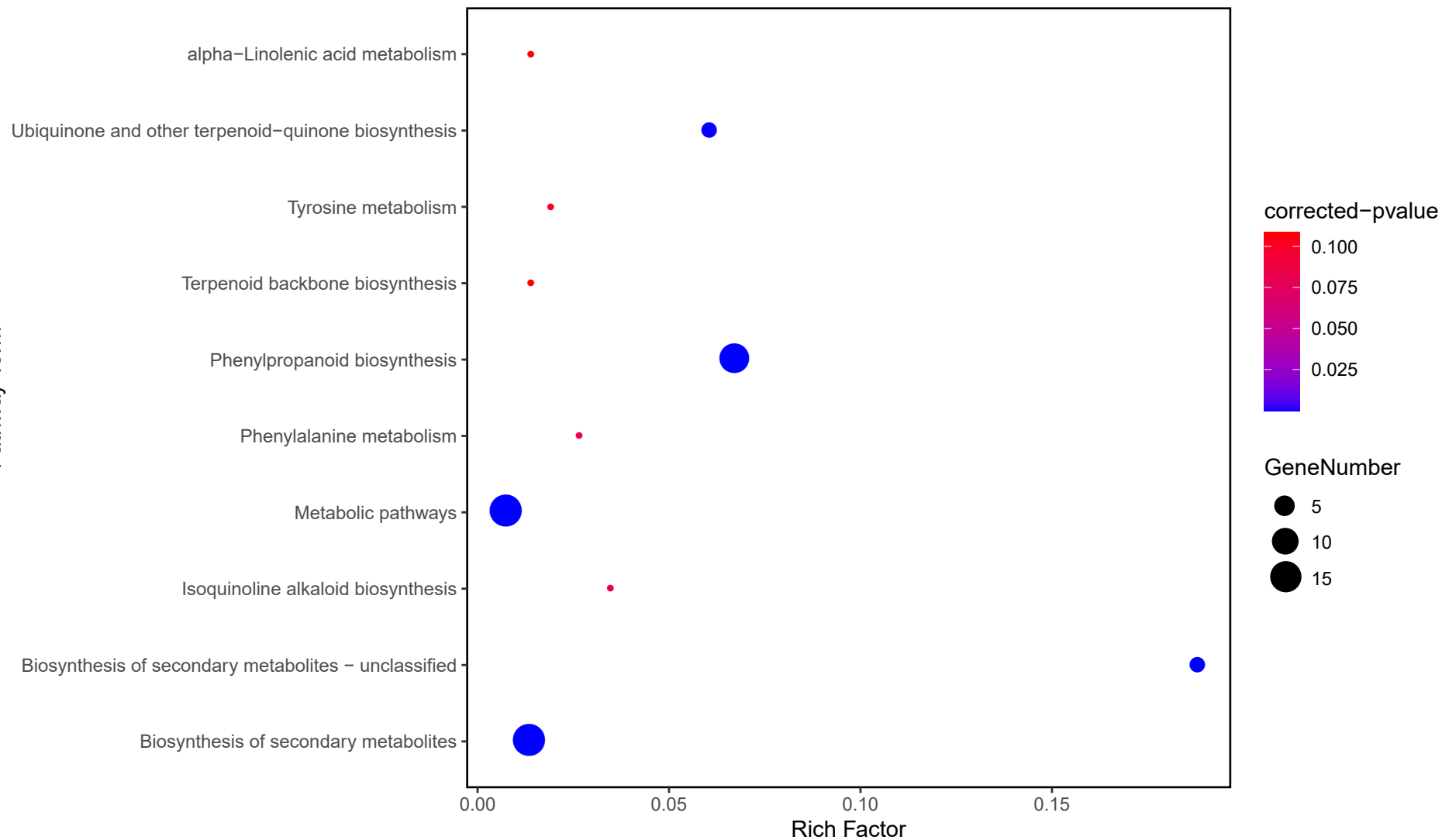

Supplement: Web_Material_uhag042 [file web_material_uhag042.zip › Supplementary Fig. 5.pdf]

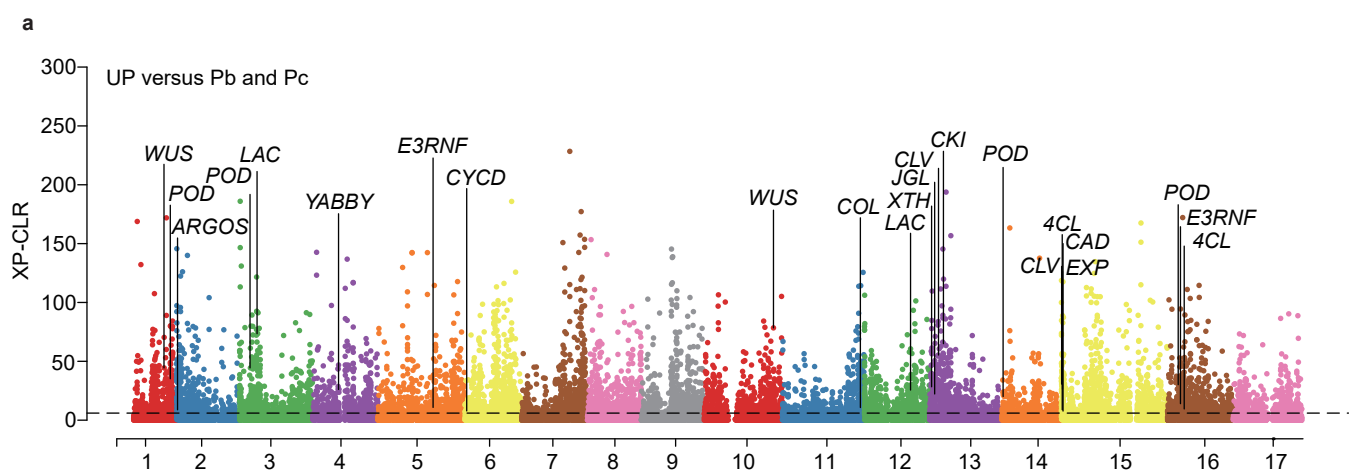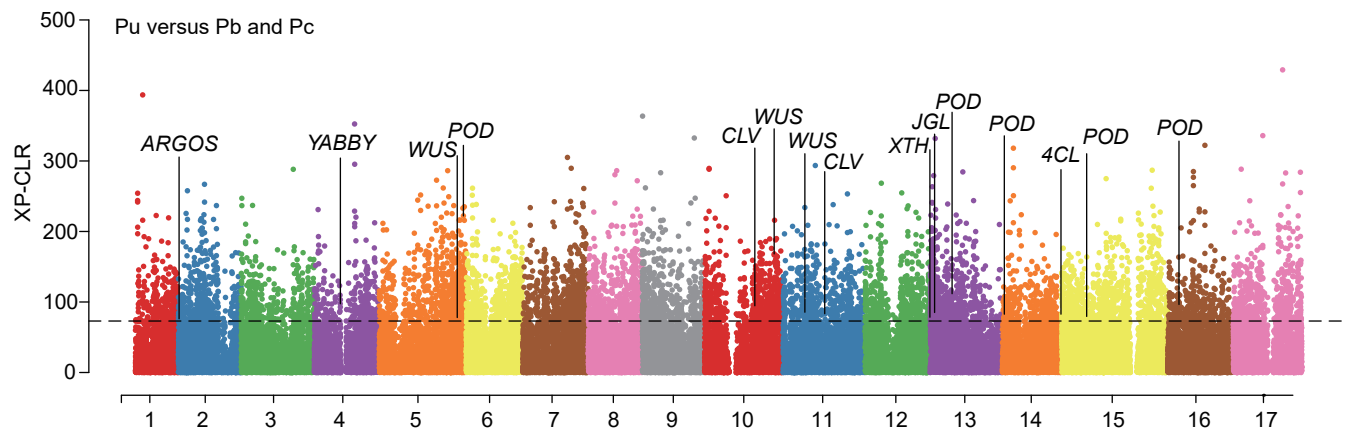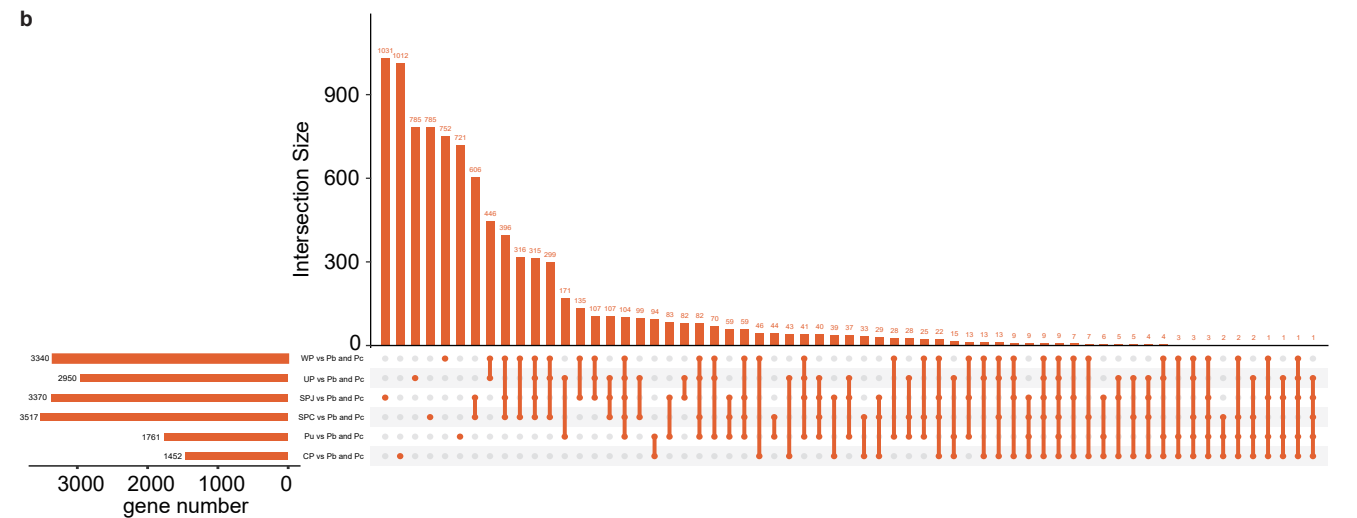

Supplement: Web_Material_uhag042 [file web_material_uhag042.zip › Supplementary Fig. 6.pdf]

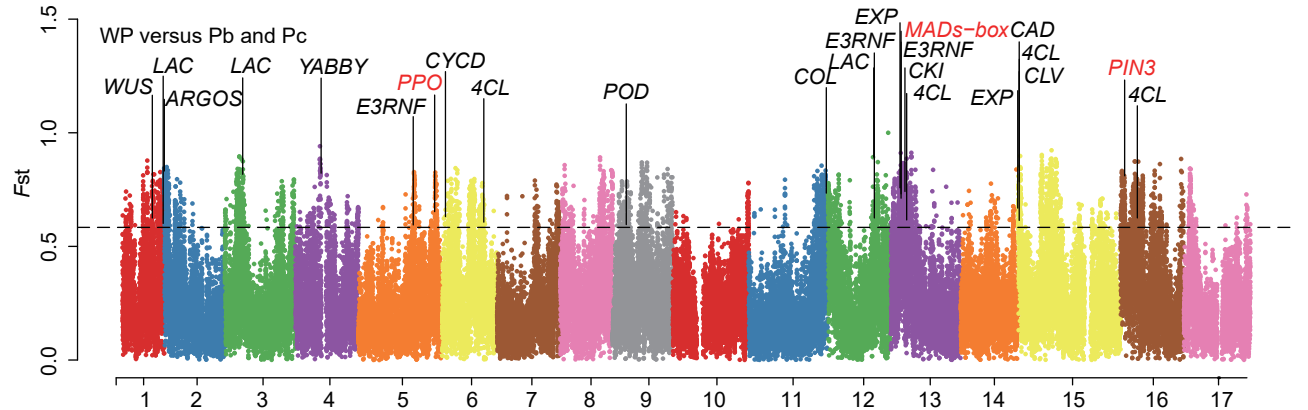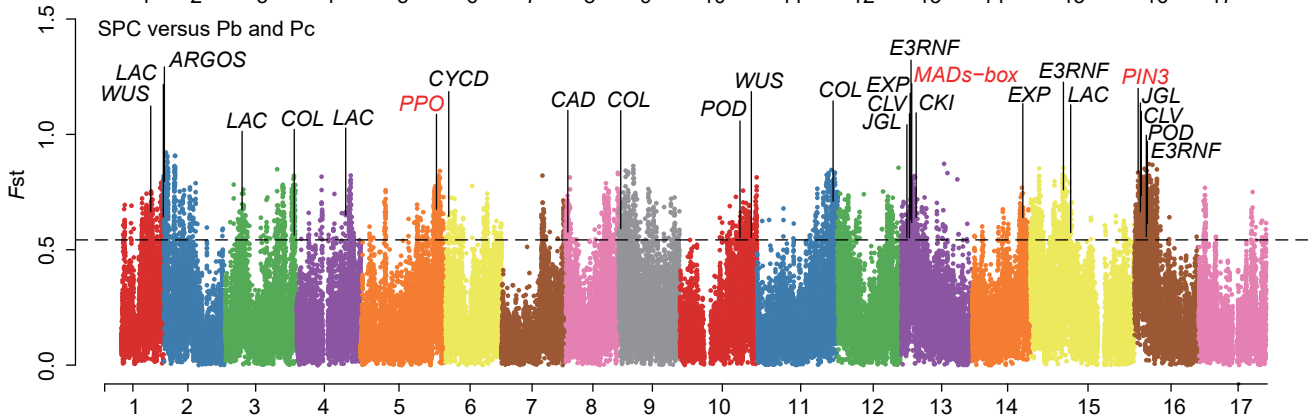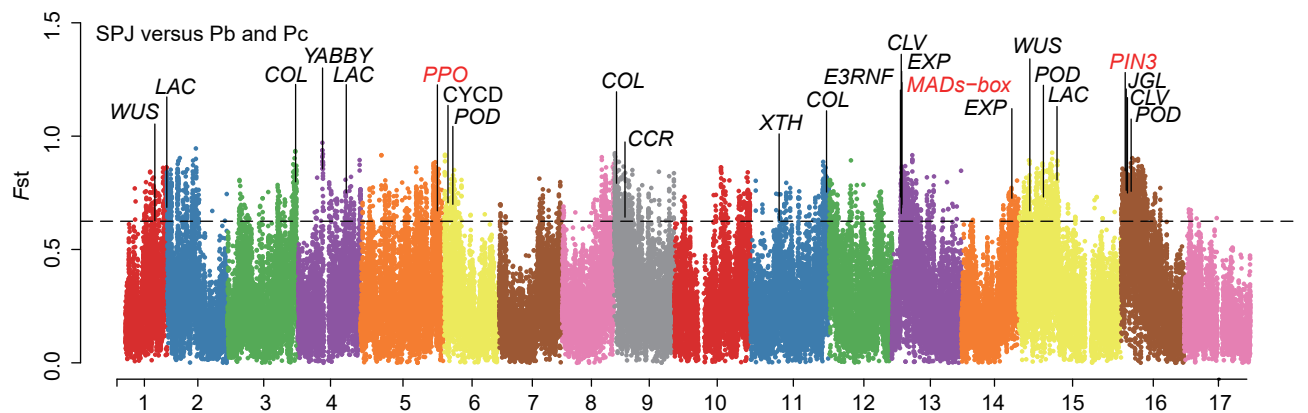

Supplement: Web_Material_uhag042 [file web_material_uhag042.zip › Supplementary Fig. 7.pdf]

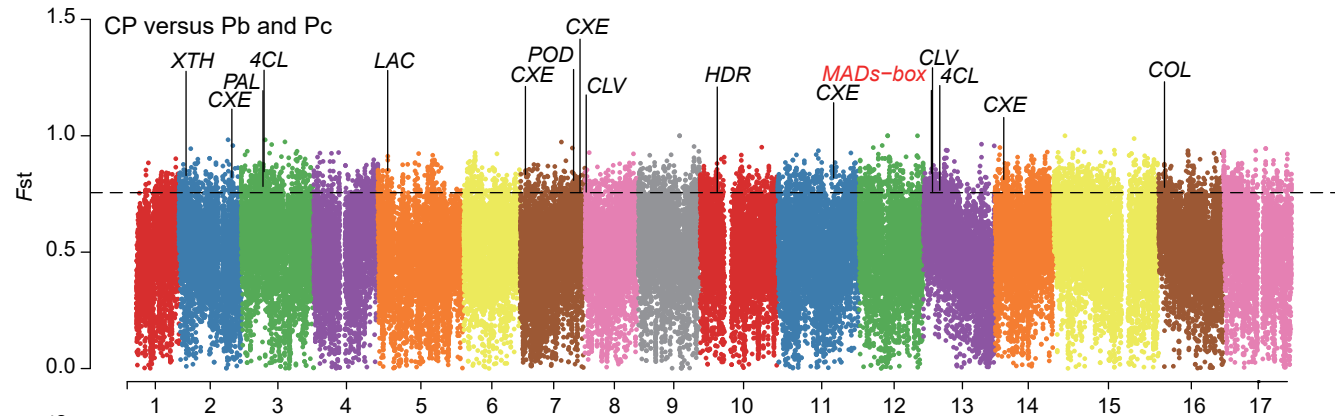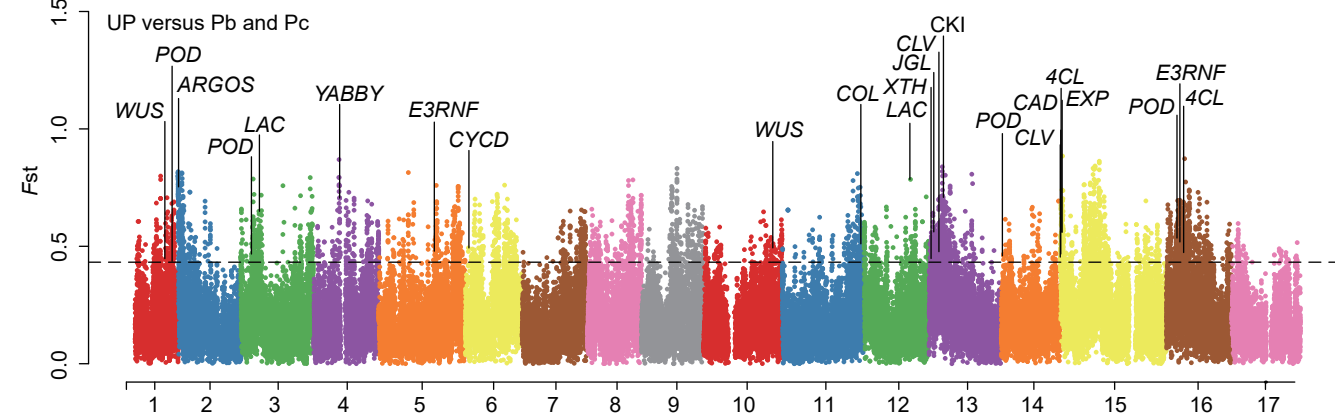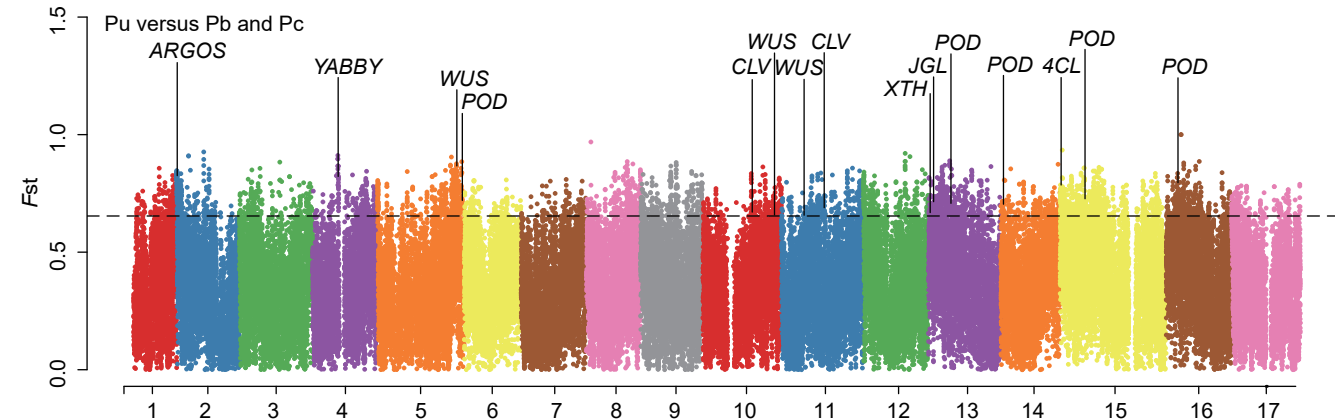

Supplement: Web_Material_uhag042 [file web_material_uhag042.zip › Supplementary Fig. 8.pdf]

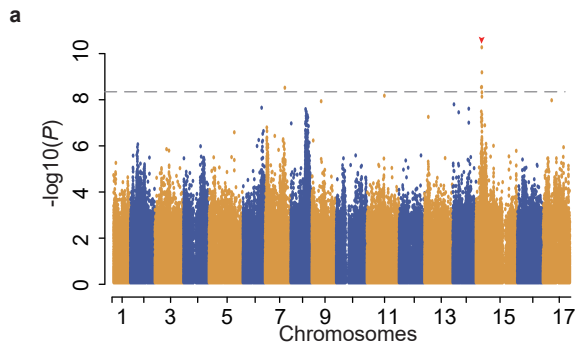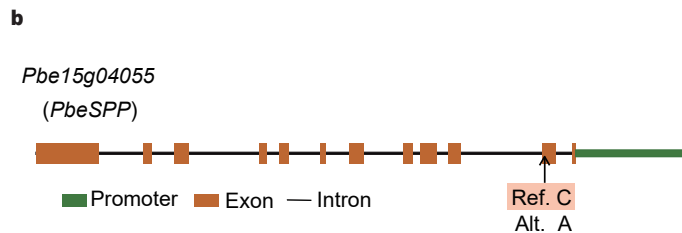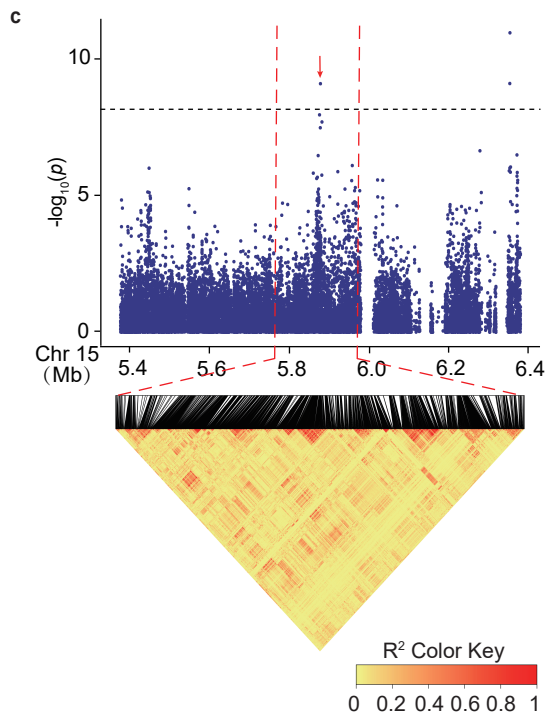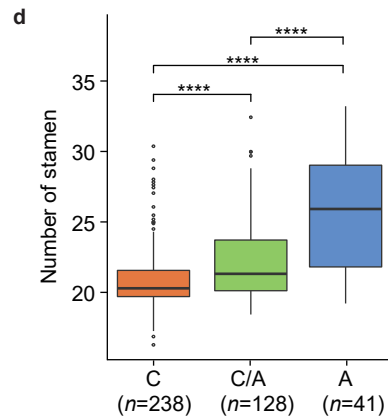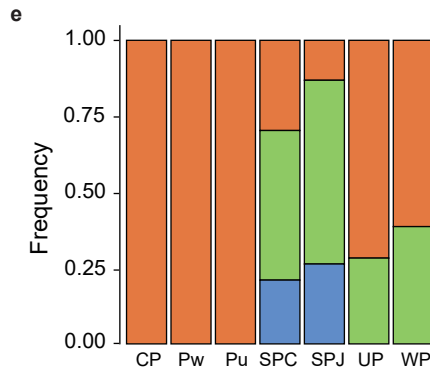

Supplement: Web_Material_uhag042 [file web_material_uhag042.zip › Supplementary Fig. 9.pdf]

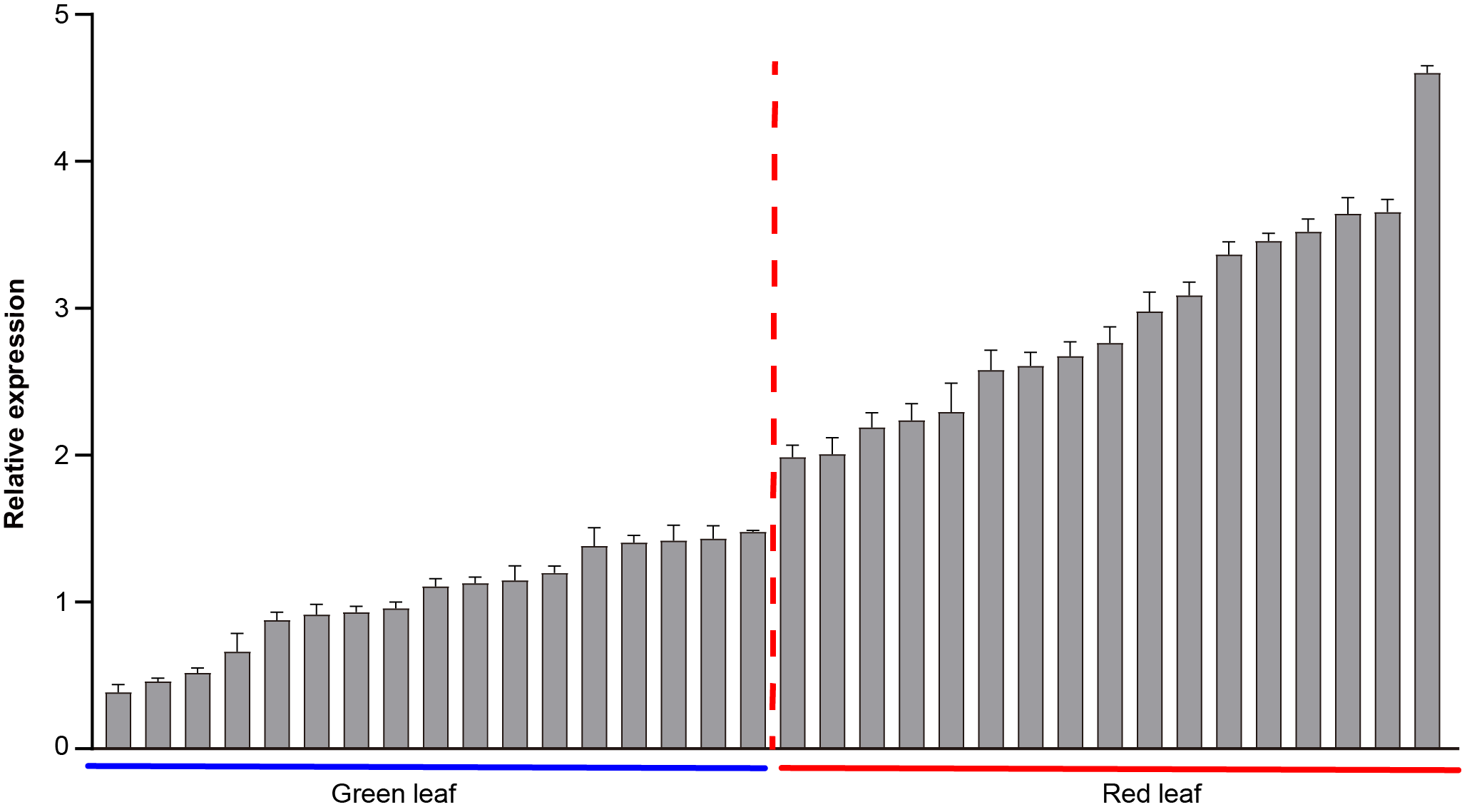

Supplement: Web_Material_uhag042 [file web_material_uhag042.zip › Supplementary Fig. 10.png]

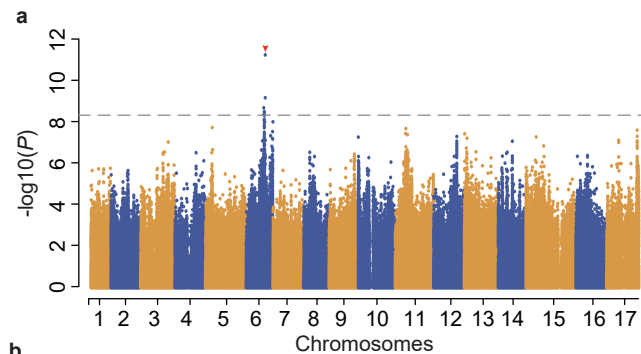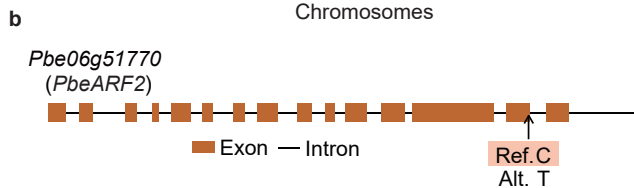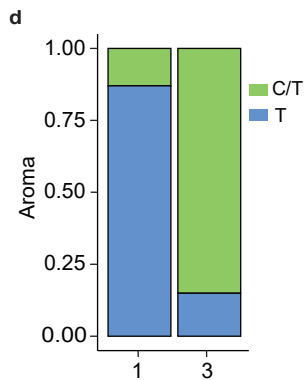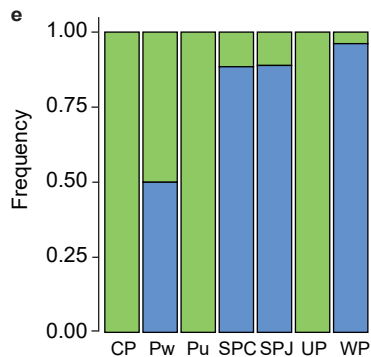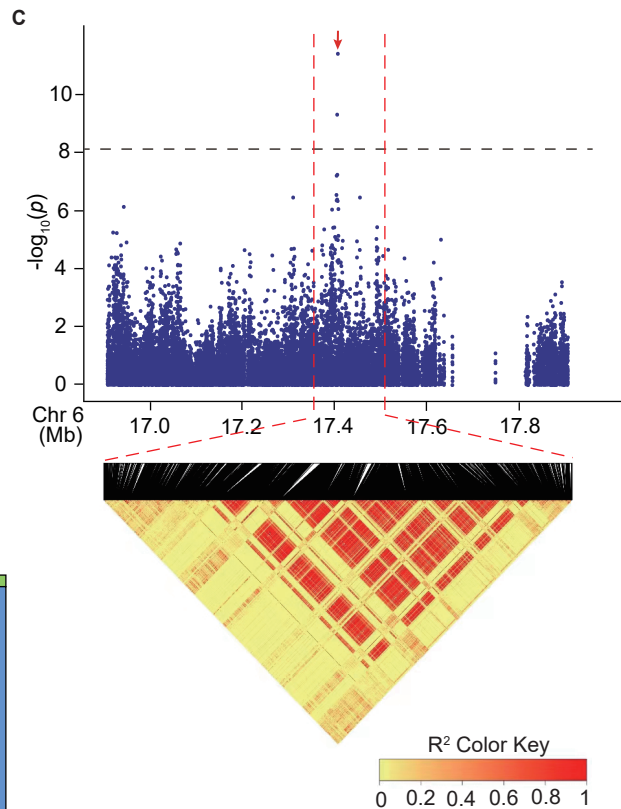

Supplement: Web_Material_uhag042 [file web_material_uhag042.zip › Supplementary Fig. 11.pdf]

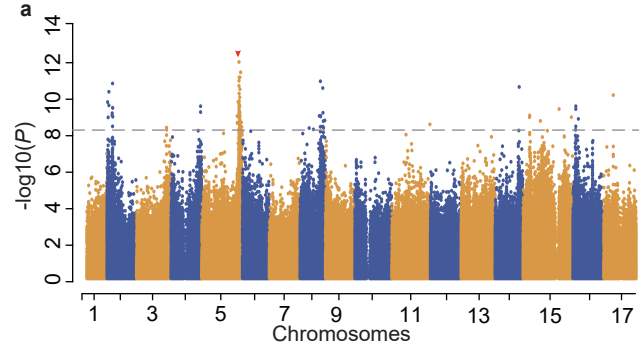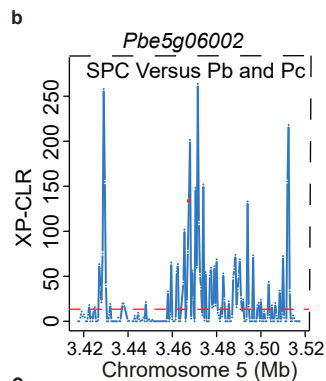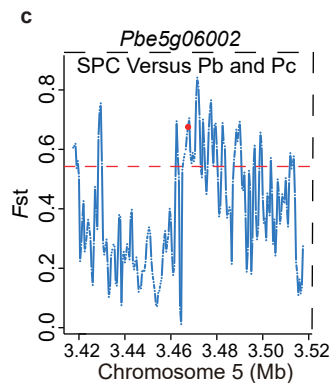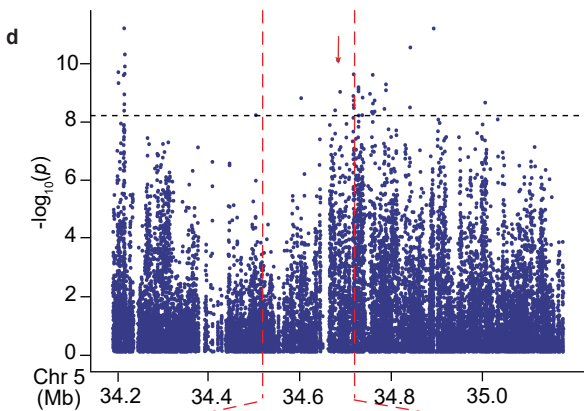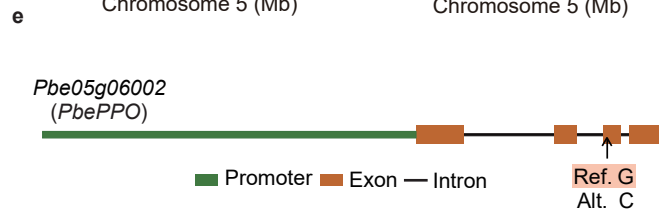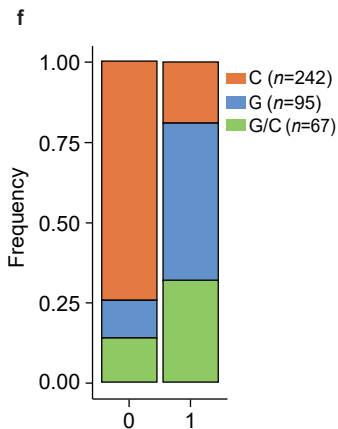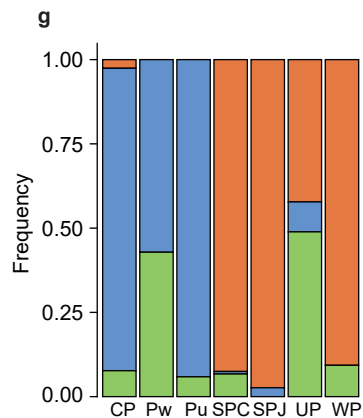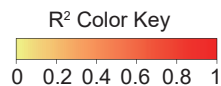

Supplement: Web_Material_uhag042 [file web_material_uhag042.zip › Supplementary Fig. 12.pdf]

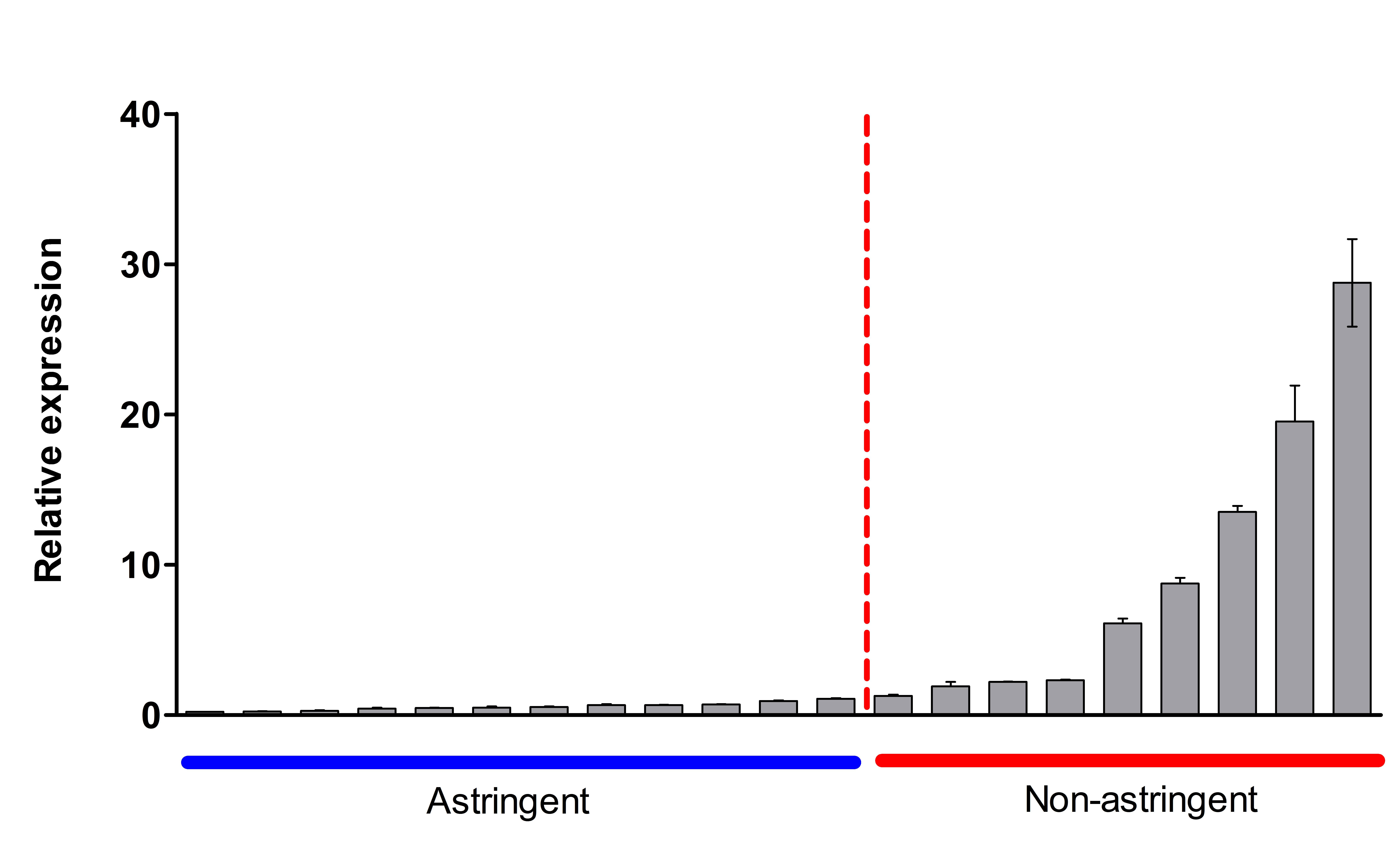

Supplement: Web_Material_uhag042 [file web_material_uhag042.zip › Supplementary Fig. 13.jpg]

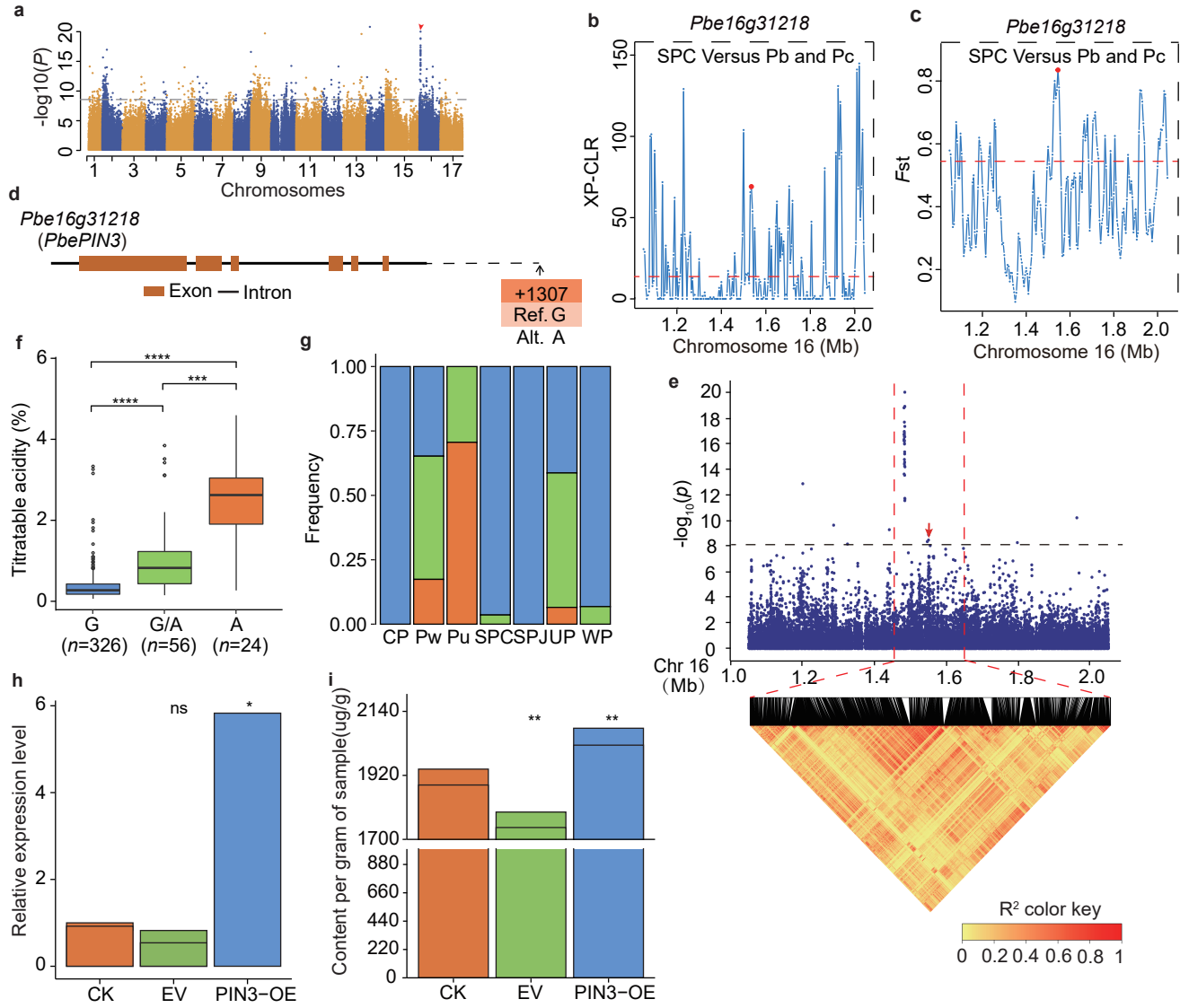

Supplement: Web_Material_uhag042 [file web_material_uhag042.zip › Supplementary Fig. 14.pdf]

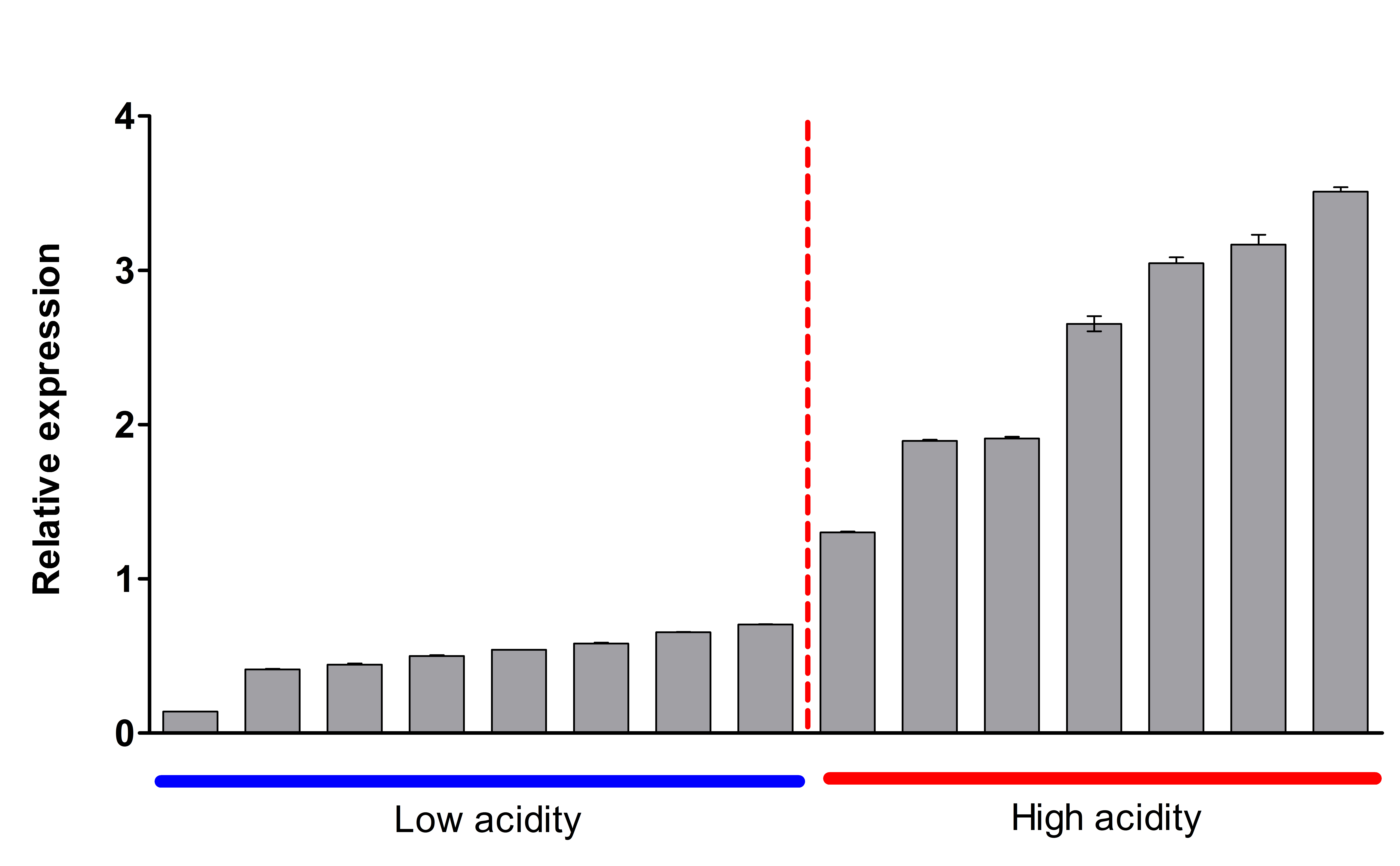

Supplement: Web_Material_uhag042 [file web_material_uhag042.zip › Supplementary Fig. 15.jpg]

**a**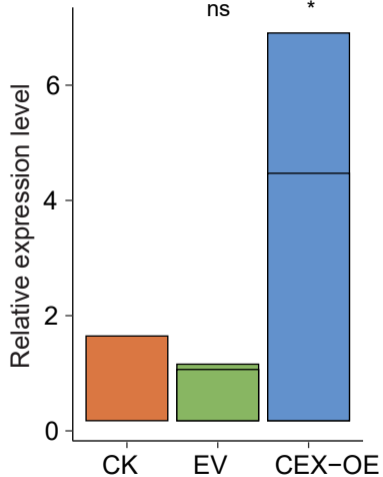**b**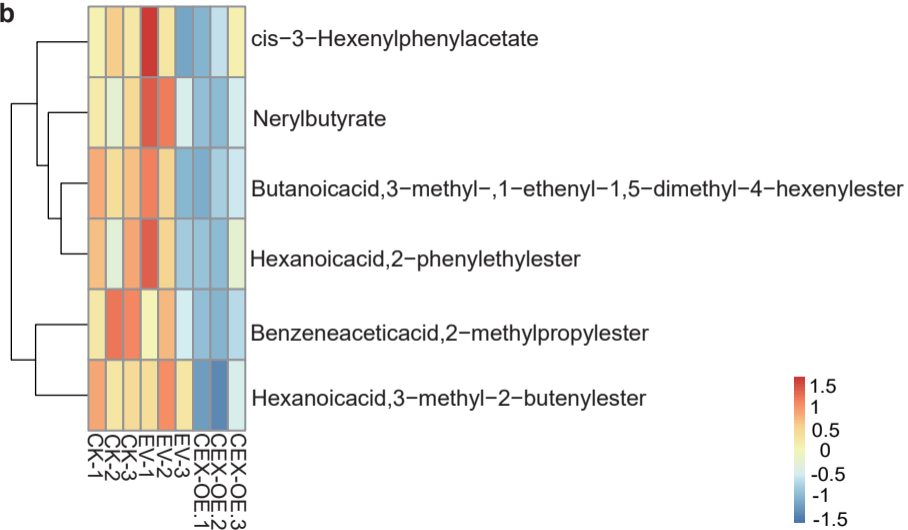

Supplement: Web_Material_uhag042 [file web_material_uhag042.zip › Supplementary Fig. 16.pdf]

**a**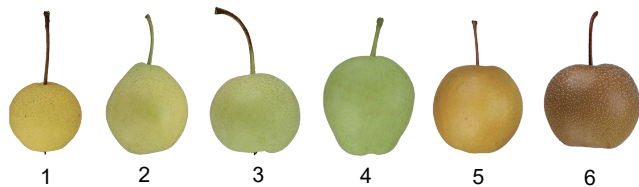**b**

*Pbe08g54210*  
(*PbeMYB38*)

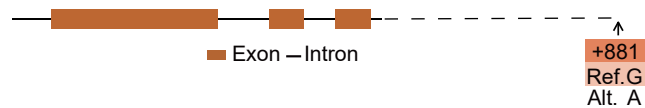**d**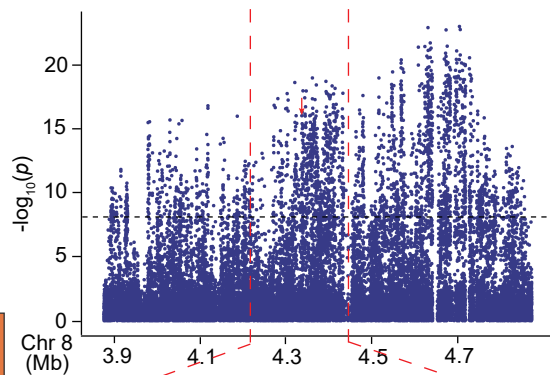**f**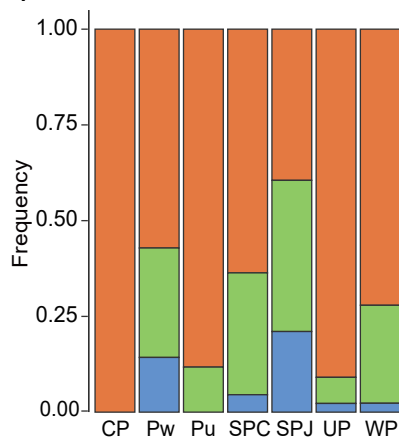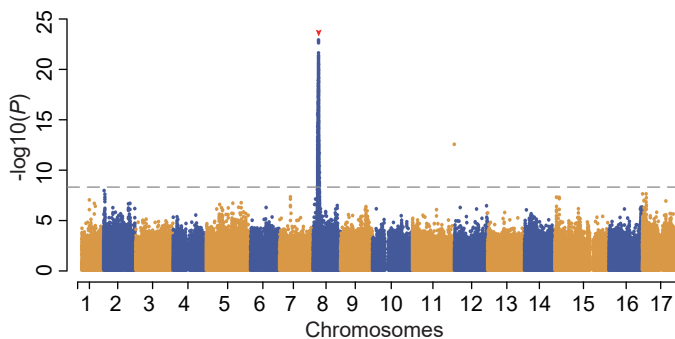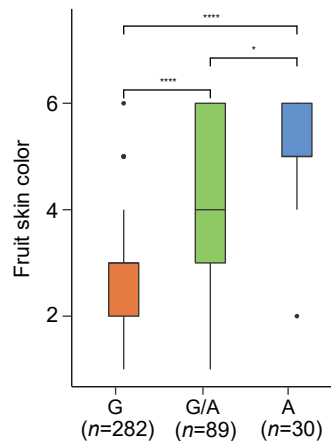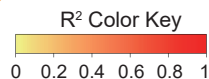

Supplement: Web_Material_uhag042 [file web_material_uhag042.zip › Supplementary Fig. 17.pdf]

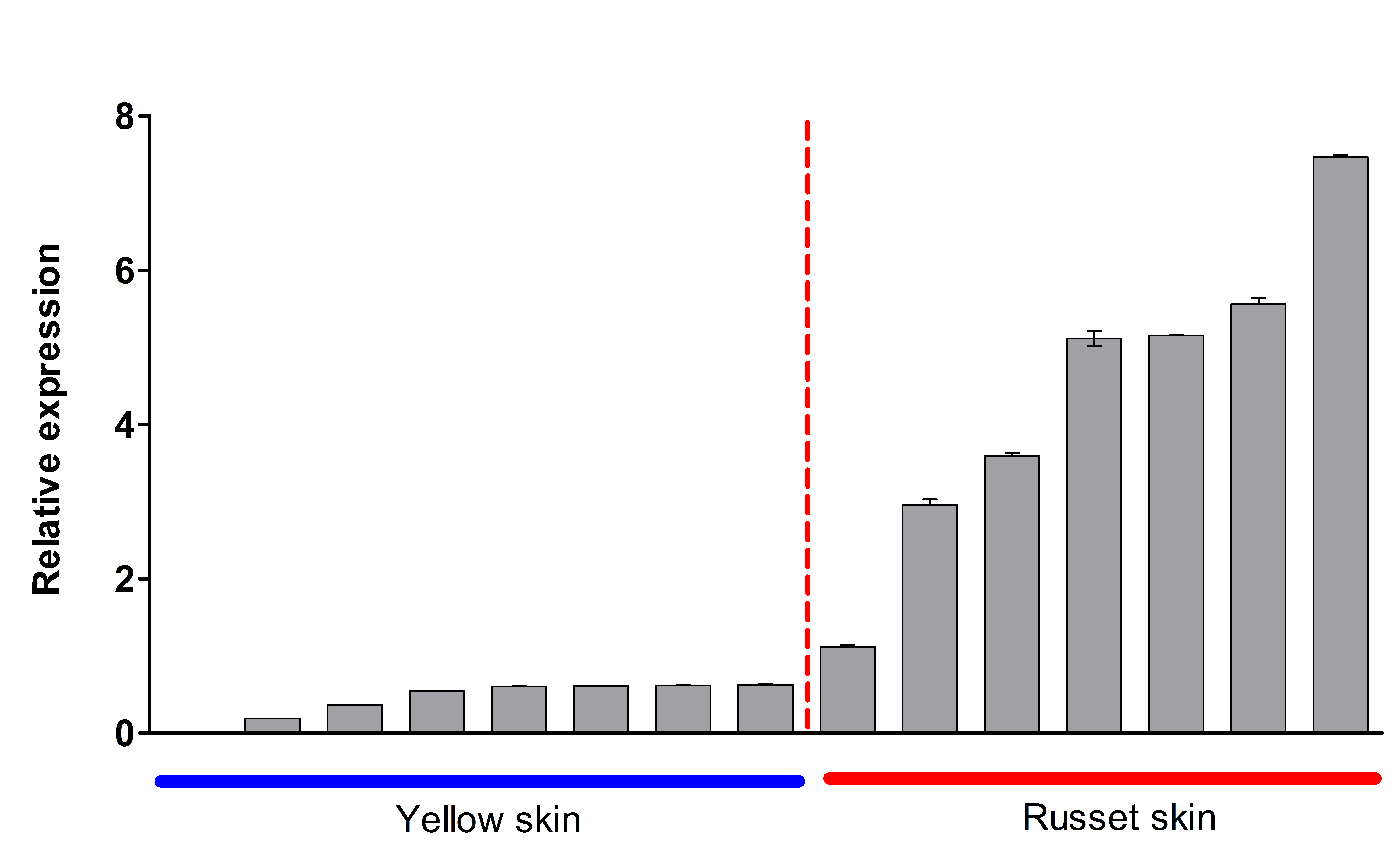

Supplement: Web_Material_uhag042 [file web_material_uhag042.zip › Supplementary Fig. 18.jpg]
